# Supplementary material for: The Gateway from Near into Remote Oceania: New Insights from Genome-Wide Data
Source: Mol Biol Evol. 2018 Jan 2;35(4):871–86. doi: 10.1093/molbev/msx333 (PMC5889034; doi:10.1093/molbev/msx333)
Supplement: Supplementary Data [file msx333_supp.pdf]

Table.S1 Populations included in the study

Population	N	Language	Lat/Long	Provenance
Australia				
Australian	3	Australian	-13/143	Lazaridis et al 2014
Australian_WGA	5	Australian	-19.64/134.19	Qin and Stoneking 2015
Island of New Guinea				
Papuan	16	Papuan	-4/143	Lazaridis et al 2014
Papuan_Eastern_Highlands	13	Papuan	-6.15/143.66	Qin and Stoneking 2015
Papuan_Huli	5	Papuan	-6.15/143.66	Qin and Stoneking 2015
Papuan_Mendi_Kewa	5	Papuan	-6.15/143.66	Qin and Stoneking 2015
Papuan_Central_Province	6	Papuan	-9.08/146.9	Qin and Stoneking 2015
Papuan_Gulf_Province	4	Papuan	-7.64/142.58	Qin and Stoneking 2015
Island of New Britain				
Baining_Marabu	10	Papuan	-4.63/152.3	Skoglund et al 2016
Tolai	20	Austronesian	-4.48/152.1	Skoglund et al 2016
Sulka	18	Papuan	-4.5/152.33	Skoglund et al 2016
Baining_Malasait	5	Papuan	-4.47/151.9	Skoglund et al 2016
Kove	18	Austronesian	-5.47/148.95	Skoglund et al 2016
Mengen	10	Austronesian	-5.1/151.4	Skoglund et al 2016
Nakanai_Bileki	10	Austronesian	-5.75/150.8	Skoglund et al 2016
Mamusi	20	Austronesian	-5.87/151.1	Skoglund et al 2016
Ata	8	Papuan	-5.58/150.89	Skoglund et al 2016
Kol_New_Britain	2	Papuan	-5.38/151.63	Skoglund et al 2016
Melamela	10	Austronesian	-5/151.25	Skoglund et al 2016
Mamusi_Paleabu	6	Austronesian	-5.95/150.9	Skoglund et al 2016
Mangseng	6	Austronesian	-5.93/150.7	Skoglund et al 2016
Nakanai_Loso	7	Austronesian	-5.48/150.8	Skoglund et al 2016
Lavongai	15	Austronesian	-2.57/150.43	Skoglund et al 2016
Island of New Ireland				
Nailik	9	Austronesian	-2.98/151.52	Skoglund et al 2016
Madak	9	Austronesian	-3.1/151.7	Skoglund et al 2016
Notsi	9	Austronesian	-3.05/151.65	Skoglund et al 2016
Tigak	10	Austronesian	-2.57/150.83	Skoglund et al 2016
Kuot_Lamalaua	4	Papuan	-3/151.5	Skoglund et al 2016
Kuot_Kabil	10	Papuan	-3.07/151.7	Skoglund et al 2016
Mussau	10	Austronesian	-1.58/149.73	Skoglund et al 2016
Manus	2	Austronesian	-2.08/147	Skoglund et al 2016
Bougainville Island				
Buka	8	Austronesian	-5.42/154.67	Skoglund et al 2016
Teop	10	Austronesian	-5.85/155.18	Skoglund et al 2016
Saposa	9	Austronesian	-5.58/154.67	Skoglund et al 2016
Bougainville_South	2	Papuan	-6.6/155.5	Skoglund et al 2016
Nasioi	10	Papuan	-6.48/155.83	Skoglund et al 2016

Western Solomon Islands				
Ranongga	6	Austronesian	-8.09/156.42	Qin and Stoneking 2015
Choiseul	7	Austronesian	-7.04/156.57	Qin and Stoneking 2015
Kolombangara	5	Austronesian	-7.99/156.93	Qin and Stoneking 2015
Vella_Lavella	6	Papuan	-7.76/156.37	Qin and Stoneking 2015
Isabel	8	Austronesian	-7.96/157.82	Qin and Stoneking 2015
Eastern Solomon Islands				
Russell	4	Papuan	-9.78/156.09	Qin and Stoneking 2015
Gela	6	Austronesian	-9/160.13	Qin and Stoneking 2015
Savo	7	Papuan	-9.14/159.81	Qin and Stoneking 2015
Malaita	6	Austronesian	-9.02/159.95	Qin and Stoneking 2015
Makira	7	Austronesian	-10.51/161.31	Qin and Stoneking 2015
Santa_Cruz	7	Austronesian	-10.76/163.71	Qin and Stoneking 2015
Polynesian Outliers				
Ontong_Java	7	Austronesian	0.98/147.93	Qin and Stoneking 2015
Tikopia	6	Austronesian	-12.09/159.77	Qin and Stoneking 2015
RenBel*	7	Austronesian	-11.66/159.99	Qin and Stoneking 2015
Polynesia				
Tongan	10	Austronesian	-21.19/-175.18	Qin and Stoneking 2015
Indonesia				
Borneo	9	Austronesian	0.18/110.16	Qin and Stoneking 2015
Semende	10	Austronesian	3.58/98.67	Qin and Stoneking 2015
Philippines				
Mamanwa1	5	Austronesian	9.76/125.51	Qin and Stoneking 2015
Mamanwa	13	Austronesian	9.76/125.51	Qin and Stoneking 2015
Taiwan				
Atayal	11	Austronesian	24.61/121.3	Lazaridis et al 2014
Atayal1	6	Austronesian	24.61/121.3	Qin and Stoneking 2015
Ami	10	Austronesian	22.84/121.19	Lazaridis et al 2014
Ami1	9	Austronesian	22.84/121.19	Qin and Stoneking 2015
East Asia				
Cambodian	10	Austroasiatic	12/105	Lazaridis et al 2014
Han	44	Sino-Tibetan	39/116	Lazaridis et al 2014
Kinh	20	Austroasiatic	10.46/106.42	Lazaridis et al 2014
Thai	22	Tai-Kadai	13.45/100.28	Lazaridis et al 2014
India				
Mala	15	Dravidian	18.67/78.17	Lazaridis et al 2014
Tiwari	15	Indo-European	21.9/83.4	Lazaridis et al 2014
Kharia	15	Austroasiatic	25.77/82.73	Lazaridis et al 2014
Lodhi	14	Austroasiatic	25.45/78.62	Lazaridis et al 2014
Vishwabrahmin	15	Indo-European	16.3/80.48	Lazaridis et al 2014
Onge	17	Andamanese	12.5/92.75	Lazaridis et al 2014

South Siberia				
Tubalar	31	Turkic	51.13/87	Lazaridis et al 2014
Altaian	7	Turkic	51.9/85.95	Lazaridis et al 2014
Tuvinian	10	Turkic	50.26/95.16	Lazaridis et al 2014
Western Eurasia				
French	29	Indo-European	46/2	Lazaridis et al 2014
English	10	Indo-European	51.19/0.73	Lazaridis et al 2014
Spanish	53	Indo-European	41.4/-4.5	Lazaridis et al 2014

*a combined sample from the islands of Renell and Bellona

Table.S2 Results of the F3 test for admixture

Source1	Source2	f3	std.err	Z-score
Papuan Gulf Province				
Papuan	Atayal	-0.034699	0.0009	-38.542
Papuan	Ami	-0.034871	0.000913	-38.174
Papuan	Han	-0.031995	0.000848	-37.713
Papuan	Tongan	-0.024171	0.000861	-28.073
Papuan_Eastern_Highlands	Tongan	-0.024814	0.000904	-27.454
Papuan_Mendi_Kewa	Tongan	-0.02457	0.000915	-26.866
Papuan Central Province				
Papuan_Eastern_Highlands	Ami	-0.043534	0.000766	-56.813
Papuan	Ami	-0.042953	0.000769	-55.85
Papuan_Eastern_Highlands	Atayal	-0.041848	0.00079	-52.955
Papuan_Eastern_Highlands	Tongan	-0.025736	0.000746	-34.497
Papuan	Tongan	-0.024912	0.000735	-33.878
Papuan_Mendi_Kewa	Tongan	-0.025157	0.000756	-33.272
Papuan				
Papuan_Eastern_Highlands	Sulka	-0.001484	0.000364	-4.083
Papuan_Eastern_Highlands	Kove	-0.001433	0.000369	-3.884
Papuan_Eastern_Highlands	Mengen	-0.00123	0.00037	-3.322
Tolai				
Baining_Malasait	Ami	-0.012025	0.000956	-12.574
Baining_Malasait	Han	-0.01041	0.000857	-12.143
Baining_Malasait	Atayal	-0.011771	0.000975	-12.068
Baining_Marabu	Tongan	-0.006881	0.000715	-9.631
Baining_Marabu	Isabel	-0.006171	0.000655	-9.427
Baining_Marabu	RenBel	-0.006551	0.000794	-8.256
Sulka				
Baining_Marabu	Ami	-0.008469	0.000892	-9.498
Baining_Malasait	Ami	-0.009222	0.00098	-9.409
Baining_Malasait	Atayal	-0.009049	0.000979	-9.243
Baining_Marabu	Tongan	-0.004663	0.000731	-6.381
Baining_Marabu	RenBel	-0.004817	0.000821	-5.865
Baining_Marabu	Tikopia	-0.0039	0.000755	-5.167
Kove				
Papuan	Ami	-0.008424	0.000871	-9.675
Papuan_Eastern_Highlands	Ami	-0.007766	0.000915	-8.483
Papuan	Atayal	-0.007242	0.00088	-8.234
Mengen				
Baining_Malasait	Atayal	-0.015624	0.001025	-15.241
Baining_Malasait	Ami	-0.015106	0.001024	-14.757
Baining_Marabu	Atayal	-0.013785	0.000953	-14.468
Baining_Marabu	Tongan	-0.00904	0.000771	-11.719
Baining_Marabu	Ontong_Java	-0.007814	0.000783	-9.985
Baining_Marabu	Tikopia	-0.008146	0.000825	-9.871
Nakanai_Bileki				
Baining_Malasait	Atayal	-0.005633	0.001149	-4.904
Baining_Malasait	Ami	-0.005435	0.001152	-4.717
Baining_Marabu	Ami	-0.004101	0.001078	-3.803
Melamela				
Baining_Malasait	Ami	-0.00983	0.001125	-8.736
Baining_Malasait	Atayal	-0.00949	0.001132	-8.383
Baining_Marabu	Ami	-0.008471	0.001039	-8.152

Mangseng				
Baining_Malasait	Ami	-0.018733	0.001089	-17.203
Baining_Malasait	Atayal	-0.018509	0.00109	-16.982
Papuan	Ami	-0.0166	0.00099	-16.775
Baining_Marabu	Tongan	-0.008394	0.000912	-9.208
Baining_Marabu	RenBel	-0.008409	0.001029	-8.17
Baining_Marabu	Ontong_Java	-0.006786	0.000949	-7.151
Nailik				
Baining_Marabu	Ami	-0.014384	0.000955	-15.066
Baining_Malasait	Ami	-0.015502	0.00107	-14.488
Baining_Marabu	Atayal	-0.013599	0.000966	-14.078
Baining_Marabu	Isabel	-0.00798	0.000731	-10.912
Baining_Marabu	Tongan	-0.007859	0.000813	-9.671
Baining_Marabu	RenBel	-0.007738	0.000906	-8.54
Madak				
Baining_Malasait	Ami	-0.01242	0.001048	-11.849
Baining_Malasait	Atayal	-0.012239	0.001067	-11.47
Baining_Marabu	Ami	-0.010823	0.001001	-10.81
Baining_Marabu	Isabel	-0.005266	0.00078	-6.753
Baining_Marabu	Tongan	-0.005559	0.000868	-6.402
Baining_Marabu	RenBel	-0.005228	0.000981	-5.331
Notsi				
Baining_Marabu	Ami	-0.017449	0.000921	-18.942
Baining_Marabu	Han	-0.015433	0.000827	-18.658
Baining_Malasait	Ami	-0.01844	0.001001	-18.416
Baining_Marabu	Isabel	-0.01145	0.000689	-16.61
Baining_Marabu	Tongan	-0.011973	0.00075	-15.968
Baining_Marabu	Ontong_Java	-0.010404	0.00074	-14.053
Tigak				
Baining_Marabu	Ami	-0.008925	0.001013	-8.806
Baining_Malasait	Ami	-0.009466	0.001092	-8.668
Baining_Malasait	Atayal	-0.009118	0.001088	-8.382
Kuot_Kabil				
Baining_Malasait	Ami	-0.006149	0.001155	-5.324
Baining_Malasait	Atayal	-0.006116	0.00116	-5.272
Baining_Marabu	Ami	-0.004921	0.00103	-4.776
Lavongai				
Baining_Malasait	Ami	-0.00885	0.001052	-8.409
Baining_Malasait	Atayal	-0.008027	0.001081	-7.423
Baining_Malasait	Han	-0.00687	0.000935	-7.347
Baining_Marabu	Isabel	-0.003357	0.000745	-4.505
Mussau				
Papuan	Ami	-0.019914	0.000845	-23.561
Papuan_Eastern_Highlands	Ami	-0.019074	0.000863	-22.108
Papuan	Atayal	-0.018629	0.00088	-21.169
Baining_Marabu	Tongan	-0.005237	0.000839	-6.242
Baining_Marabu	RenBel	-0.004841	0.000976	-4.961
Baining_Marabu	Ontong_Java	-0.003222	0.000838	-3.845
Manus				
Papuan	Ami	-0.032711	0.001483	-22.064
Papuan	Atayal	-0.032204	0.001482	-21.727
Papuan	Han	-0.028703	0.001436	-19.993

	Papuan	Tongan	-0.025139	0.001403	-17.923
	Papuan	Ontong_Java	-0.023265	0.001393	-16.697
	Papuan	RenBel	-0.024726	0.0015	-16.48
Buka					
	Nasioi	Ami	-0.017533	0.00075	-23.365
	Nasioi	Atayal	-0.016744	0.000746	-22.434
	Nasioi	Tongan	-0.013918	0.000641	-21.698
	Baining_Marabu	Isabel	-0.010961	0.000687	-15.963
	Baining_Marabu	Tongan	-0.009491	0.000829	-11.455
	Baining_Marabu	RenBel	-0.010003	0.000957	-10.45
Teop					
	Nasioi	Ami	-0.003097	0.000834	-3.714
	Nasioi	Atayal	-0.002785	0.000846	-3.291
Saposa					
	Nasioi	Ami	-0.012064	0.000785	-15.361
	Nasioi	Atayal	-0.011465	0.000809	-14.18
	Nasioi	Han	-0.009645	0.000709	-13.604
	Bougainville_South	Ontong_Java	-0.007521	0.00101	-7.448
	Bougainville_South	Tikopia	-0.007743	0.001064	-7.279
	Bougainville_South	Tongan	-0.007573	0.001056	-7.171
Ranongga					
	Nasioi	Ami	-0.017742	0.000909	-19.522
	Nasioi	Atayal	-0.016842	0.000915	-18.414
	Nasioi	Han	-0.013918	0.000815	-17.072
	Nasioi	Tongan	-0.01274	0.000798	-15.961
	Nasioi	Tikopia	-0.011719	0.000827	-14.176
	Nasioi	RenBel	-0.012104	0.000878	-13.783
Choiseul					
	Nasioi	Ami	-0.01499	0.000857	-17.489
	Nasioi	Atayal	-0.014323	0.000862	-16.619
	Nasioi	Han	-0.012217	0.00078	-15.665
	Nasioi	Tongan	-0.01032	0.000753	-13.713
	Nasioi	Tikopia	-0.009908	0.000783	-12.651
	Nasioi	Ontong_Java	-0.009117	0.000763	-11.942
Kolombangara					
	Nasioi	Ami	-0.017468	0.000963	-18.137
	Nasioi	Atayal	-0.016538	0.000955	-17.324
	Nasioi	Han	-0.012673	0.000909	-13.941
	Nasioi	Tongan	-0.009836	0.000914	-10.765
	Nasioi	Tikopia	-0.009344	0.000953	-9.808
	Nasioi	RenBel	-0.00963	0.001044	-9.223
Vella_Lavella					
	Nasioi	Ami	-0.019908	0.000793	-25.114
	Nasioi	Atayal	-0.019349	0.000802	-24.136
	Nasioi	Han	-0.016481	0.00072	-22.89
	Nasioi	Tongan	-0.014226	0.000706	-20.147
	Nasioi	Ontong_Java	-0.012905	0.000691	-18.687
	Nasioi	Tikopia	-0.014062	0.000762	-18.454
Isabel					
	Nasioi	Ami	-0.020062	0.000773	-25.968
	Nasioi	Atayal	-0.018903	0.000803	-23.545
	Nasioi	Han	-0.014328	0.00071	-20.183

Nasioi	Tongan	-0.009458	0.000749	-12.63
Nasioi	RenBel	-0.009871	0.000894	-11.039
Nasioi	Tikopia	-0.008328	0.000832	-10.012
Russell				
Nasioi	Ami	-0.01688	0.001032	-16.361
Nasioi	Atayal	-0.016922	0.001057	-16.009
Nasioi	Han	-0.013907	0.000962	-14.45
Nasioi	Tongan	-0.011538	0.000939	-12.287
Nasioi	Tikopia	-0.011053	0.001006	-10.99
Nasioi	RenBel	-0.010526	0.001058	-9.952
Gela				
Baining_Marabu	Ami	-0.024779	0.00094	-26.366
Baining_Malasait	Ami	-0.025419	0.000974	-26.103
Papuan	Ami	-0.023027	0.000901	-25.545
Nasioi	Tongan	-0.013368	0.000732	-18.268
Nasioi	Ontong_Java	-0.011295	0.0007	-16.133
Nasioi	Tikopia	-0.012559	0.000779	-16.116
Savo				
Papuan	Ami	-0.022442	0.00088	-25.514
Papuan_Eastern_Highlands	Ami	-0.022494	0.000911	-24.694
Baining_Marabu	Ami	-0.023649	0.000961	-24.599
Nasioi	Tongan	-0.012981	0.000671	-19.332
Papuan	Isabel	-0.012339	0.000663	-18.617
Papuan_Eastern_Highlands	Isabel	-0.012066	0.000688	-17.538
Malaita				
Baining_Marabu	Ami	-0.023496	0.000966	-24.312
Papuan	Ami	-0.022208	0.000918	-24.183
Papuan_Eastern_Highlands	Ami	-0.022062	0.000936	-23.558
Nasioi	Tongan	-0.013129	0.000706	-18.604
Nasioi	Tikopia	-0.012078	0.000797	-15.159
Nasioi	RenBel	-0.012599	0.000877	-14.373
Makira				
Papuan	Ami	-0.028032	0.000802	-34.952
Baining_Marabu	Ami	-0.028287	0.000836	-33.827
Papuan_Eastern_Highlands	Ami	-0.027869	0.000825	-33.778
Papuan	Tongan	-0.018597	0.000688	-27.014
Papuan_Eastern_Highlands	Tongan	-0.018682	0.000719	-25.978
Baining_Marabu	Tongan	-0.018176	0.00072	-25.26
Santa-Cruz				
Baining_Malasait	Atayal	-0.005475	0.001197	-4.574
Baining_Malasait	Ami	-0.004431	0.001216	-3.643
Baining_Marabu	Atayal	-0.003785	0.001058	-3.576
Papuan	Isabel	-0.002268	0.000742	-3.059
Ontong_Java				
Papuan_Eastern_Highlands	Ami	-0.007764	0.001098	-7.068
Papuan	Ami	-0.007417	0.001097	-6.764
Papuan_Mendi_Kewa	Ami	-0.006922	0.0011	-6.291
Tongan				
Baining_Marabu	Ami	-0.006344	0.001107	-5.732
Baining_Malasait	Ami	-0.006387	0.001165	-5.481
Papuan	Ami	-0.005632	0.001036	-5.435

figS1

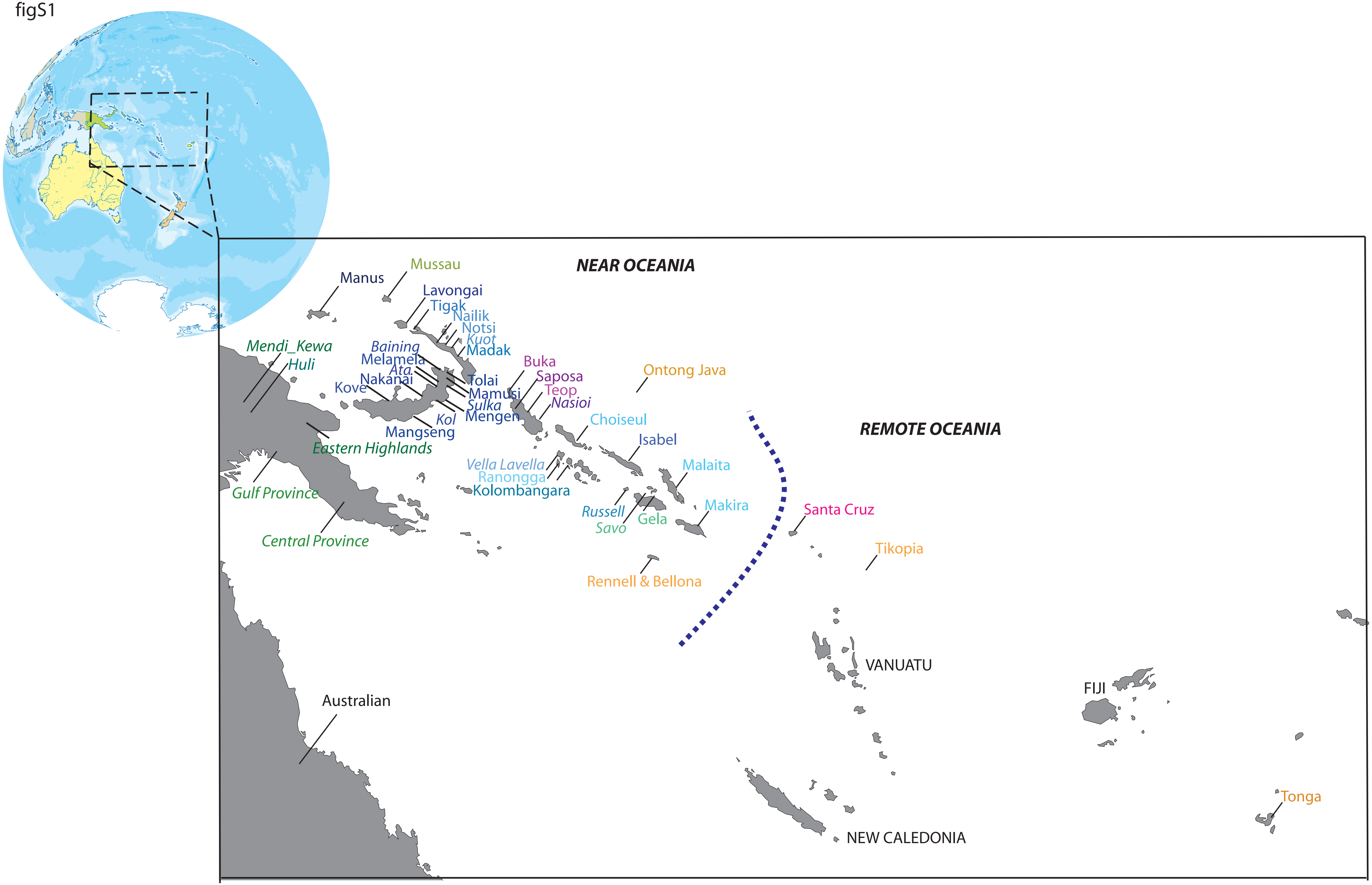

Fig.S1 Sampling locations for the populations included in the study. The curved dotted line denotes the biogeographic boundary between Near and Remote Oceania. Italicized labels indicate Papuan-speaking groups

figS2

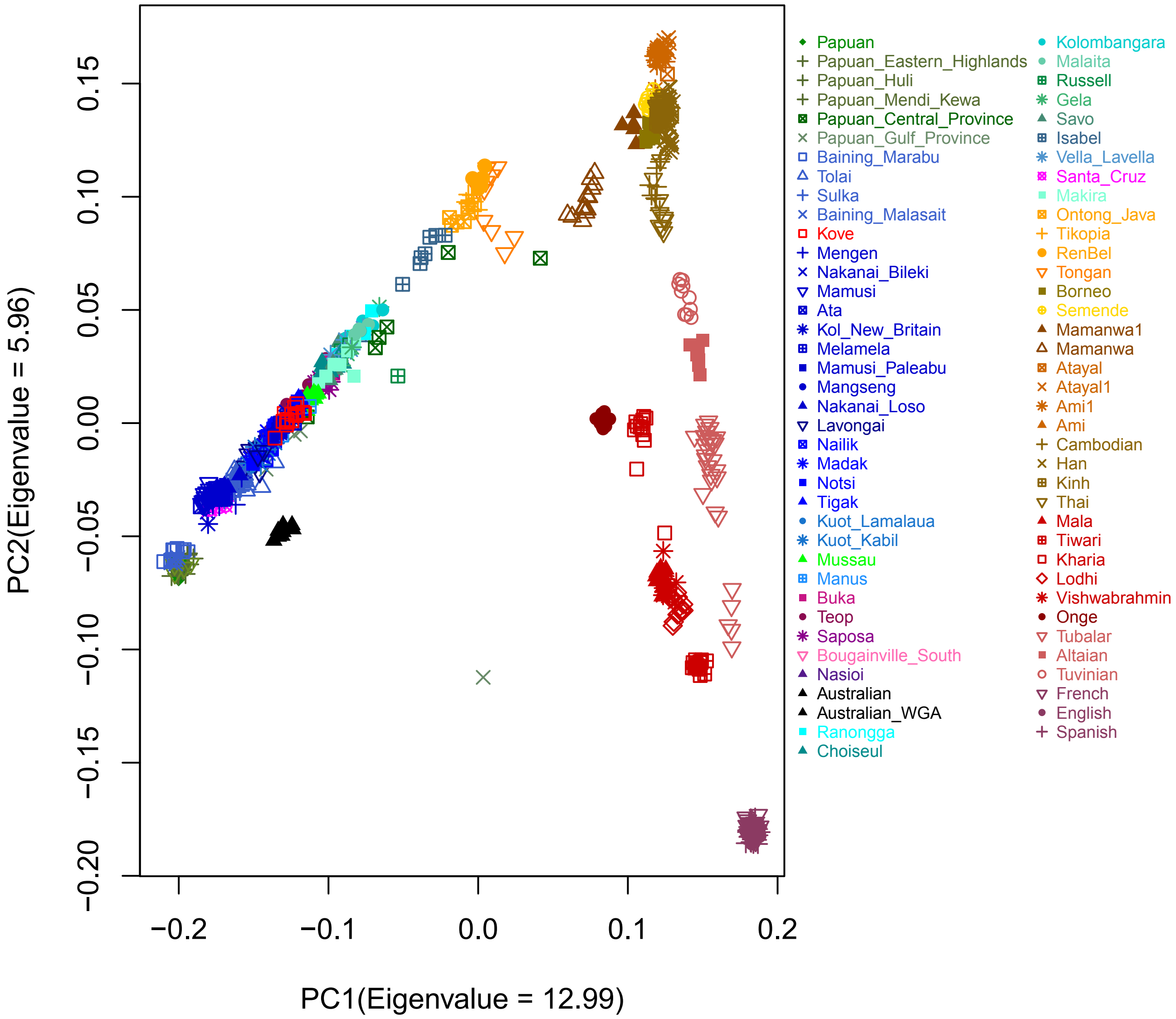

Fig.S2 Results of the PC analysis performed on the entire dataset, which includes samples from 50 populations from Near and Remote Oceania, as well as samples from Mainland and Island South East Asia, India and Western Eurasia.

figS3

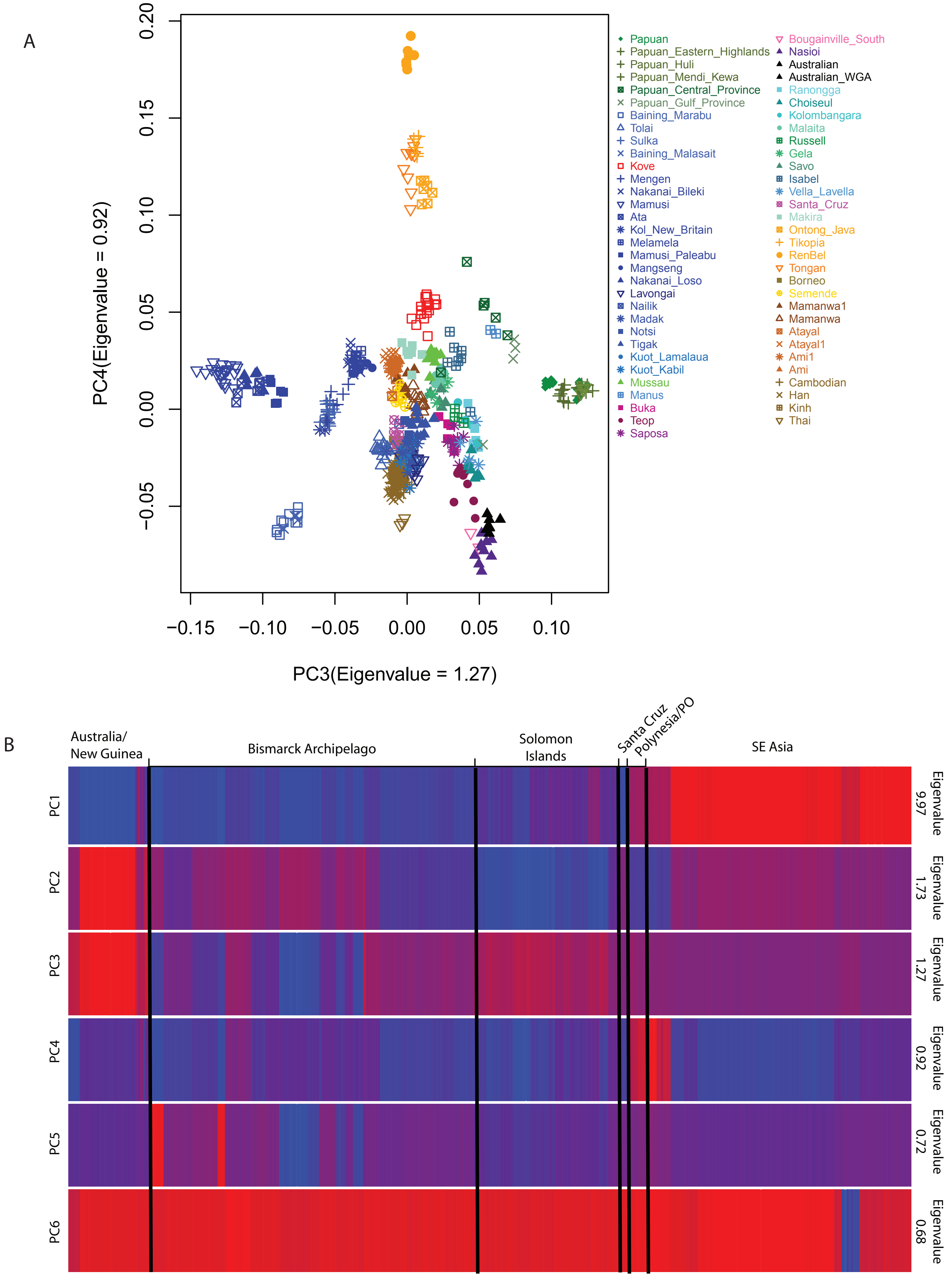

Fig.S3 Results of the PC analysis for a subset of the dataset which only includes samples from Oceania and South East Asia. (A) PC3 vs PC4 (B) Heatplot of PC loadings for each individual on each of the first 6 PC axes. The PC values for each PC have been normalized to range from 0 to 1.

figS4

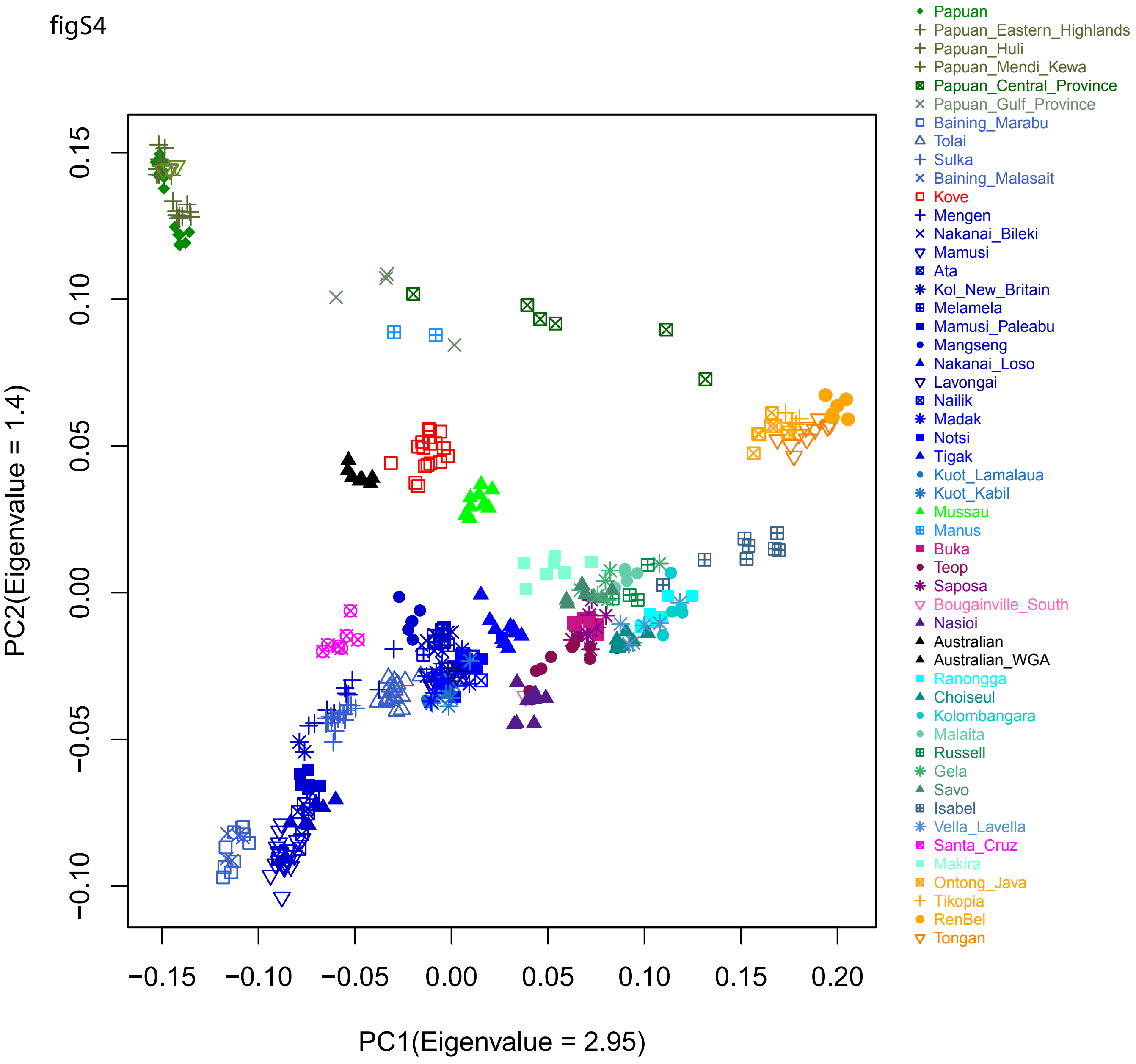

Fig.S4 Results of the PC analysis for the subset of the dataset, consisting only of samples from Near and Remote Oceania

fig5

A

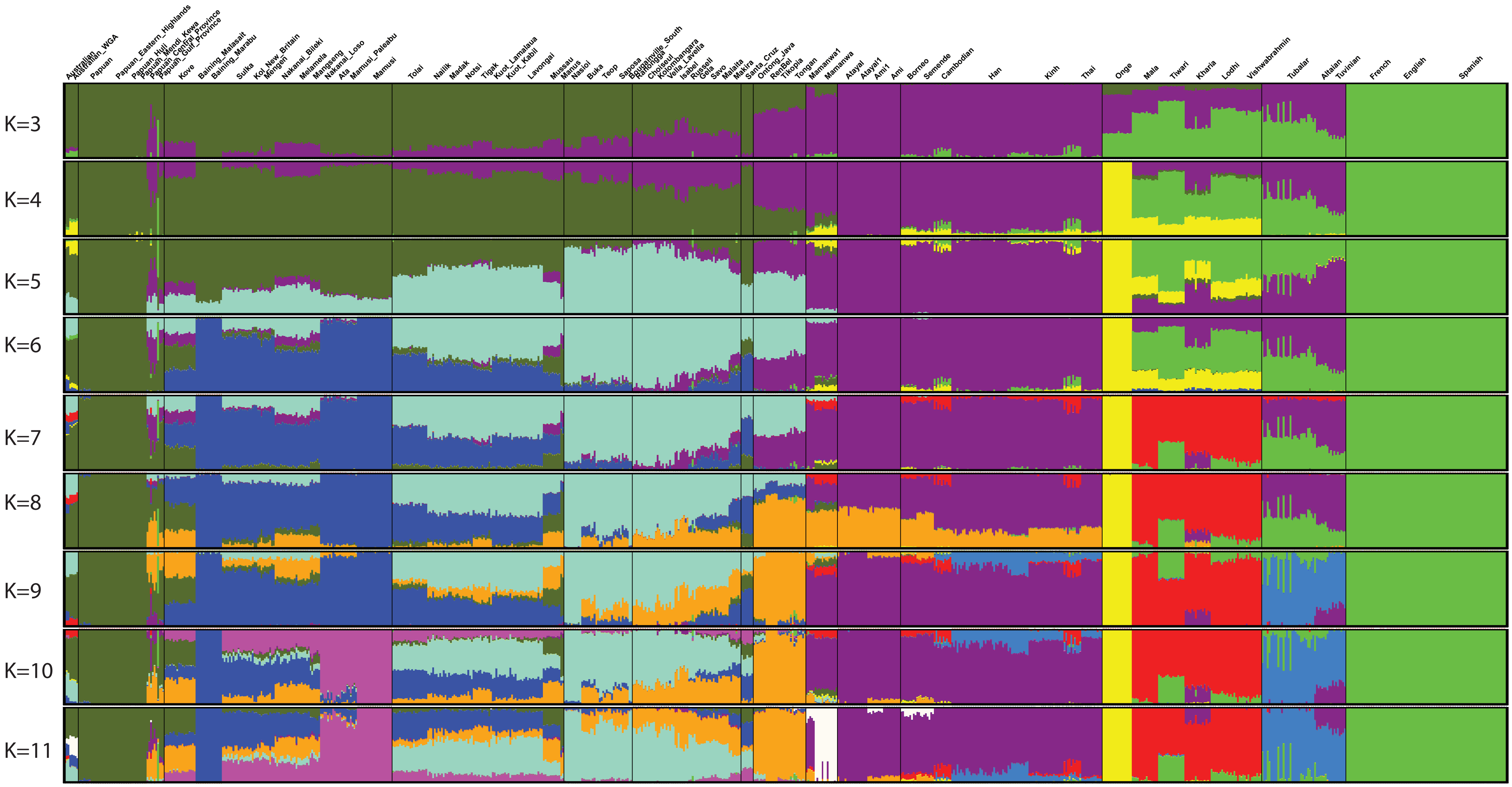

Australian
Australian_WGA
Papuan
Papuan_Eastern_Highlands
Papuan_Huli
Papuan_Mendi_Kewa
Papuan_Central_Province
Papuan_Gulf_Province
Kove
Baining_Malasait
Baining_Marabu
Sulka
Kol_New_Britain
Mengen
Nakanai_Bileki
Melamela
Mangseng
Nakanai_Loso
Ata
Mamusi_Paleabu
Mamusi
Tolai
Nailik
Madak
Notsi
Tigak
Kuot_Lamalaua
Kuot_Kabil
Lavongai
Mussau
Manus
Nasioi
Buka
Teop
Saposa
Bougainville_South
Ranongga
Choiseul
Kolombangara
Vella_Lavella
Isabel
Russell
Gela
Savo
Malaita
Makira
Santa_Cruz
Ontong_Java
RenBel
Tikopia
Tongan
Mamanwa1
Mamanwa
Atayal
Atayal1
Ami1
Ami
Borneo
Semende
Cambodian
Han
Kinh
Thai
Onge
Mala
Tiwari
Kharia
Lodhi
Vishwabrahmin
Tubalar
Altaian
Tuvinian
French
English
Spanish
K=3
K=4
K=5
K=6
K=7
K=8
K=9
K=10
K=11

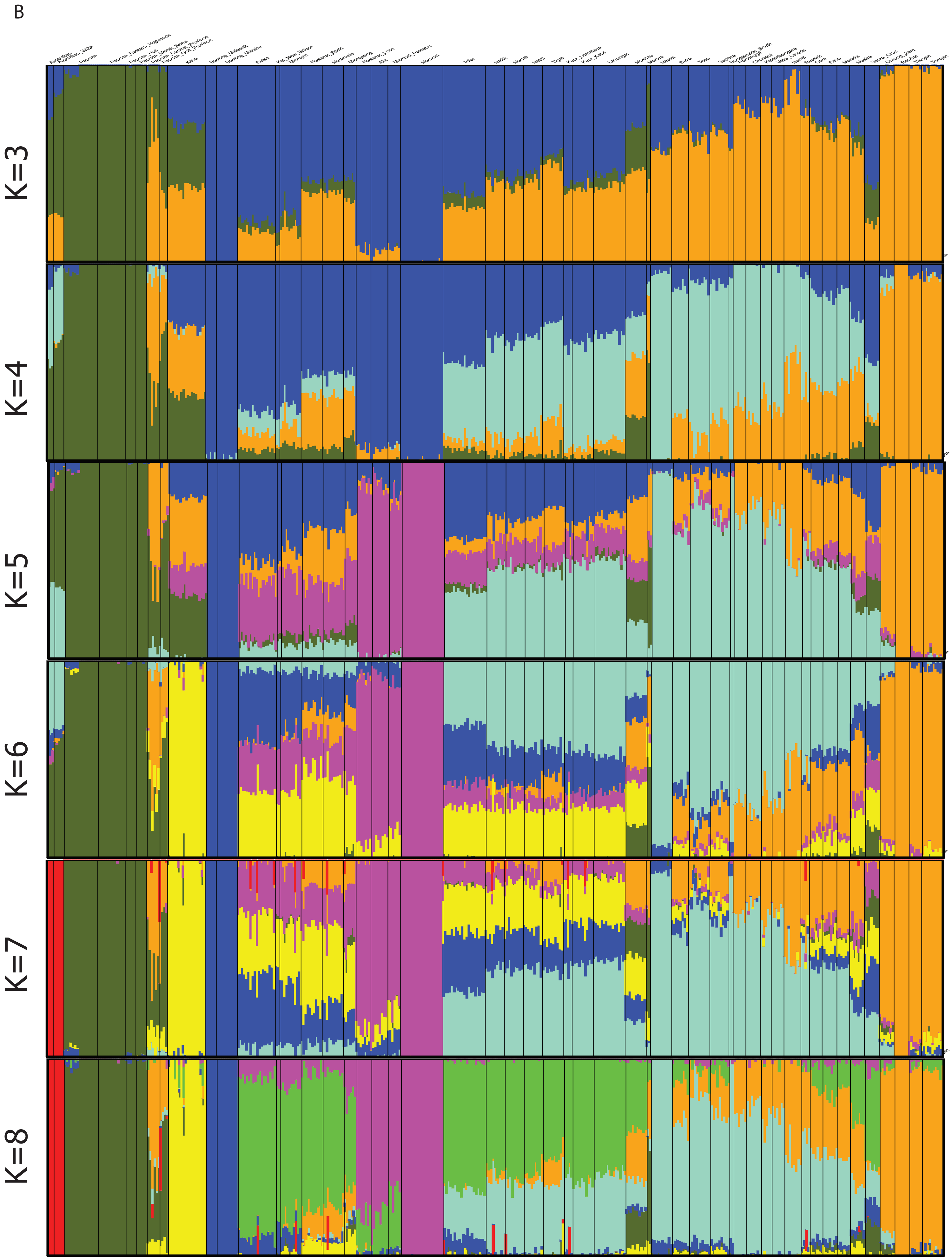

Fig.S5 Results of the ADMIXTURE analysis performed (A) on the entire dataset, (B) on the subset consisting only of samples from Oceania and South East Asia.

figS6

A

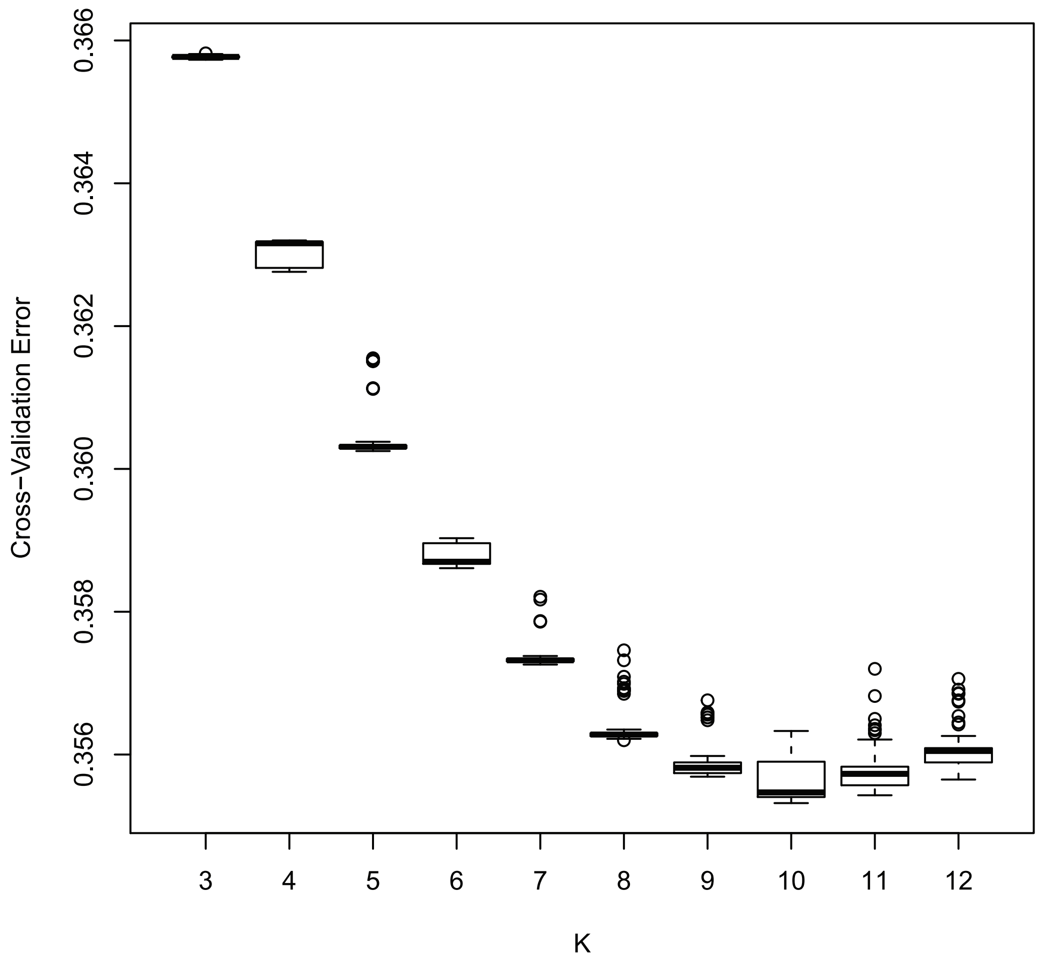

B

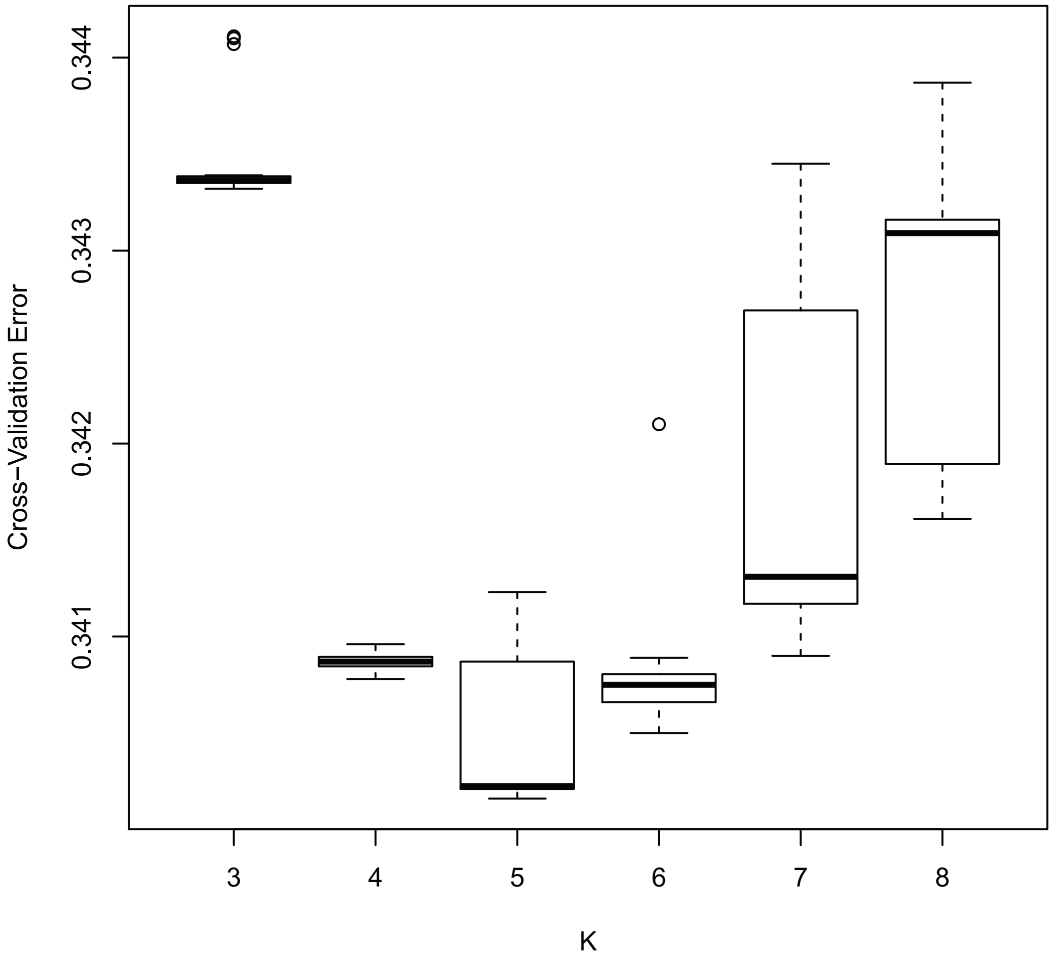

Fig.S6 Estimated cross-validation error for the ADMIXTURE runs for (A) the entire dataset, (B) the subset consisting only of samples from Oceania and South East Asia.

figS7

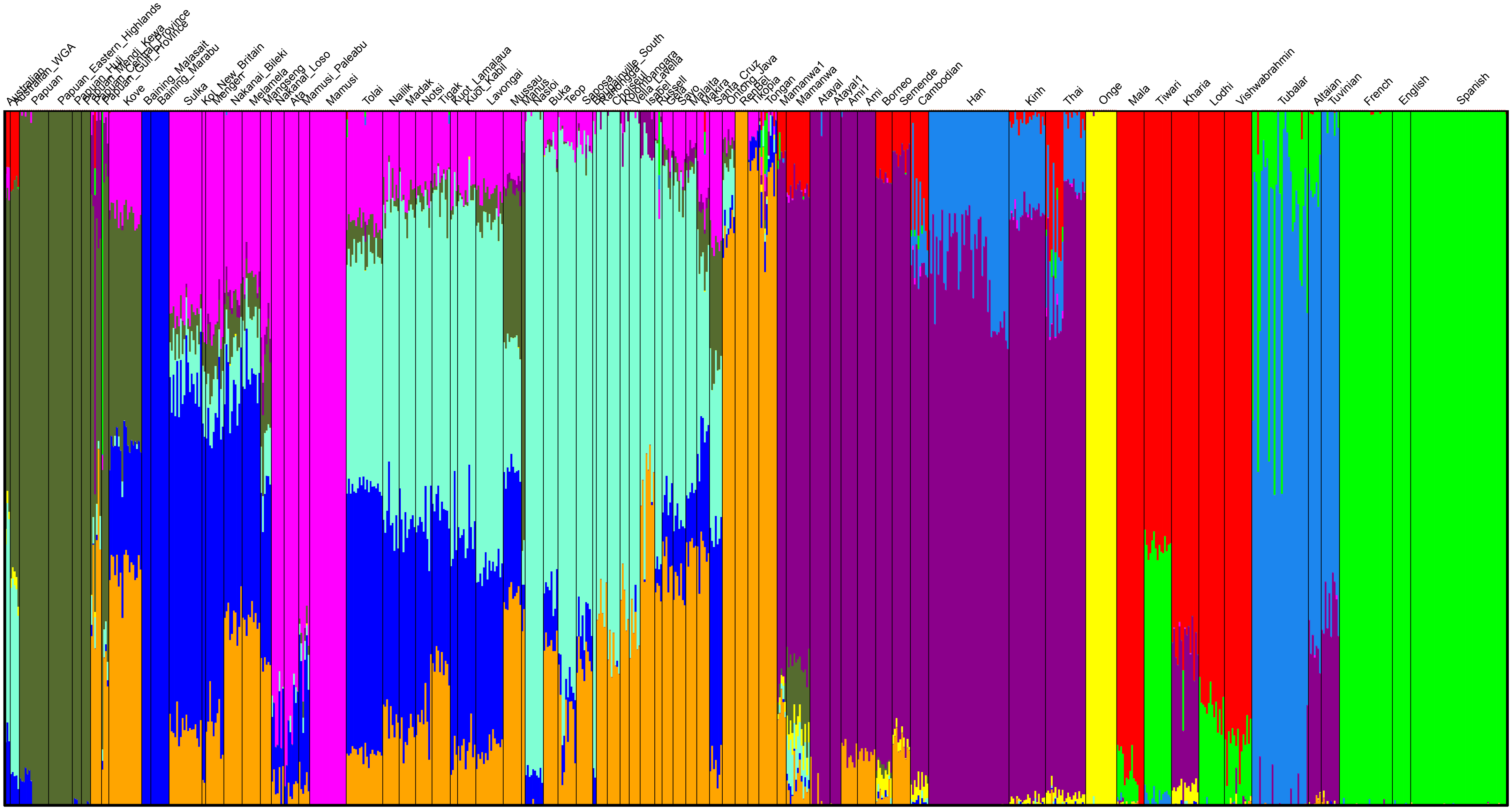

Fig.S7 Results of the ADMIXTURE analysis performed on the entire dataset. Admixture estimates presented are for K=10, which had the lowest cross-validation error (Fig.S6A)

figS8

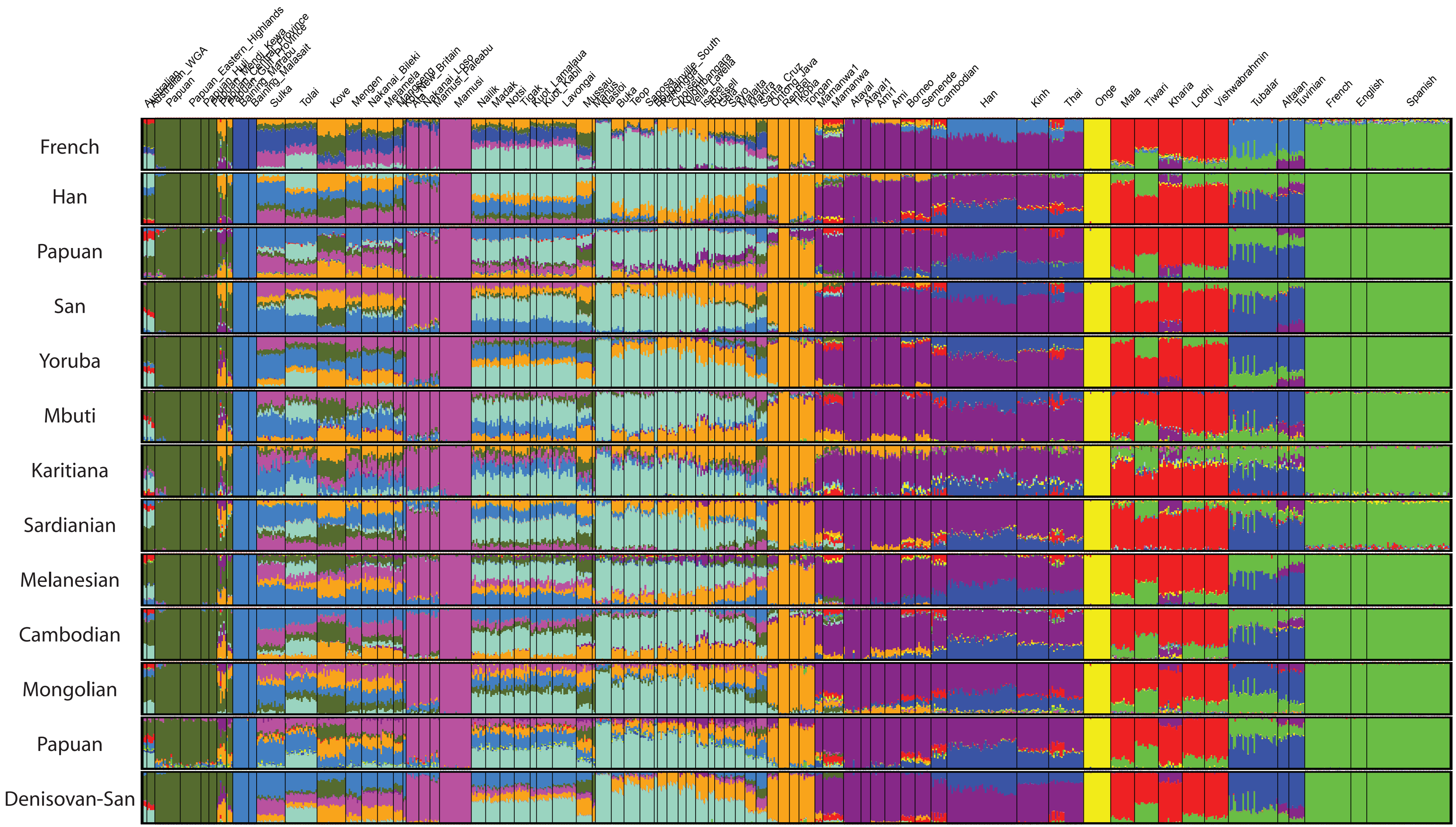

Fig.S8 ADMIXTURE results for K=10 for different ascertainment panels; the name of the panel is given on the left. In general, higher levels of Papuan ancestry (dark green component) are inferred across the Bismarcks and eastern Solomons than is inferred for these populations with the full set of markers, which is probably due to the limited resolution offered by only a subset of SNPs to differentiate between the closely related Papuan-like ancestries (blue, aqua and pink components). Interestingly, with Papuan ascertainment panels there is 15- 20% less Papuan and 5% more Indian ancestry inferred in Australia, which is supportive of previous claims of Indian gene flow to Australia (Pugach I, Delfin F, Gunnarsdottir E, Kayser M, Stoneking M. 2013. Genome-wide data substantiate Holocene gene flow from India to Australia. Proc Natl Acad Sci. 110:1803–1808.)

figS9

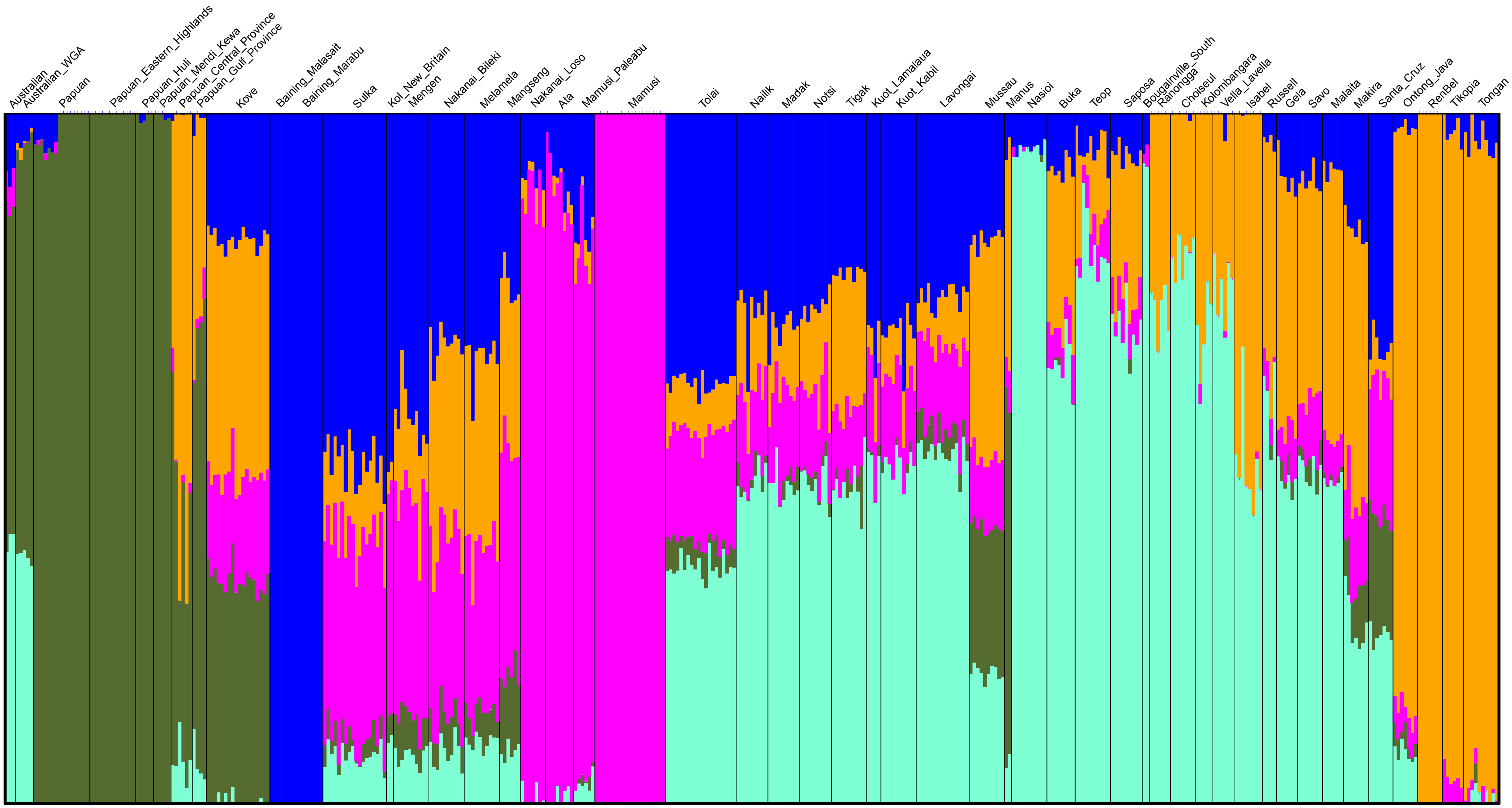

Fig.S9 Results of the ADMIXTURE analysis performed on the subset of the data which only includes samples from Oceania and South East Asia. Admixture estimates presented are for K=5, which had the lowest cross-validation error (Fig.S6B). These are the same results as those presented in Fig.2, where they are superimposed on the geographic map according to each population's sampling location.

figS10

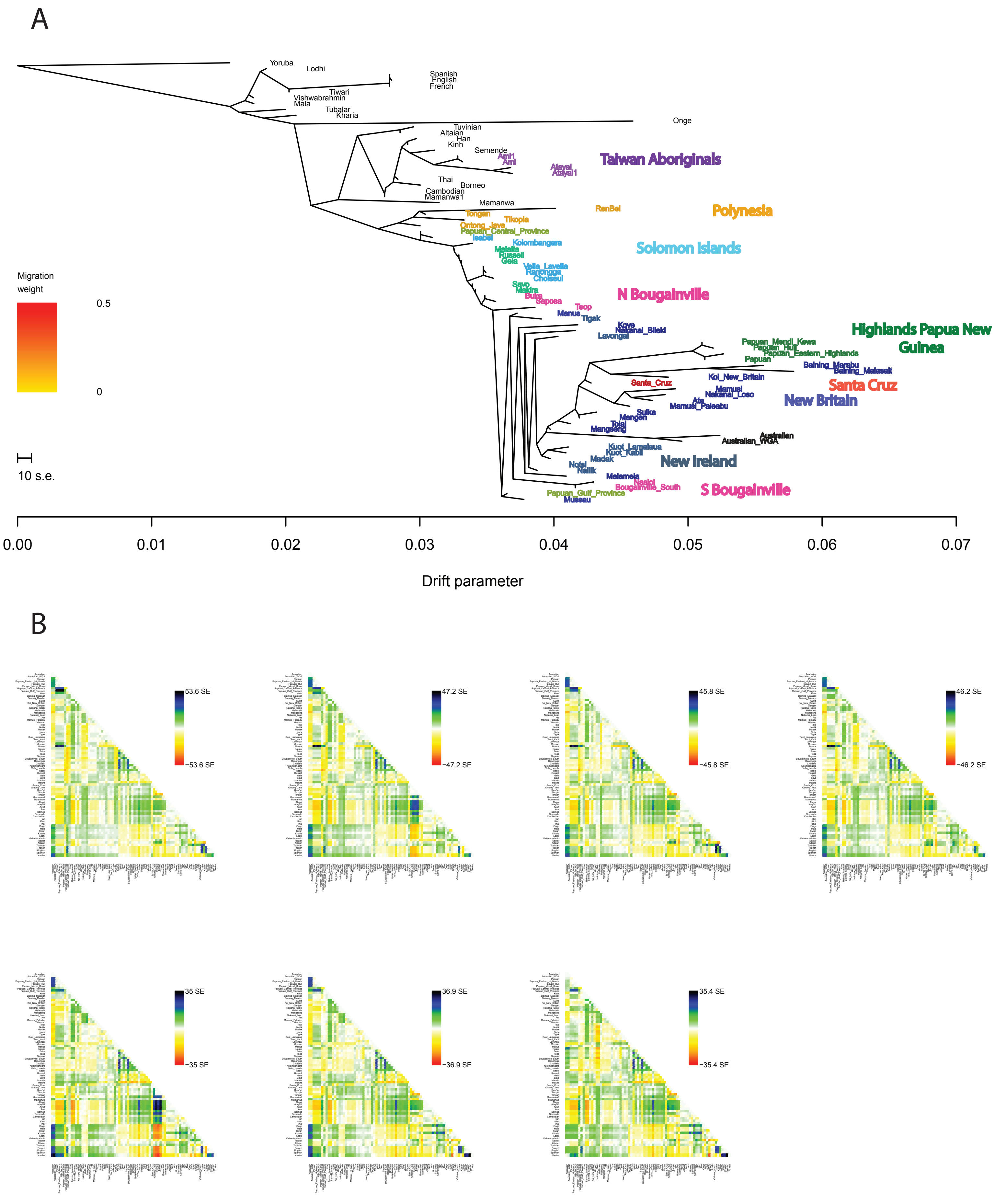

Fig.S10 TreeMix analysis on the full dataset of 75 populations, with the Yorubans added as an outgroup (A) TreeMix inferred maximum-likelihood tree (B) Residual fit from maximum likelihood trees with 1 to 7 migration edges. Positive residuals indicate pairs of populations where the model underestimates the observed covariance, while negative residuals indicate pairs of populations where the observed covariance is overestimated.

figS11

A

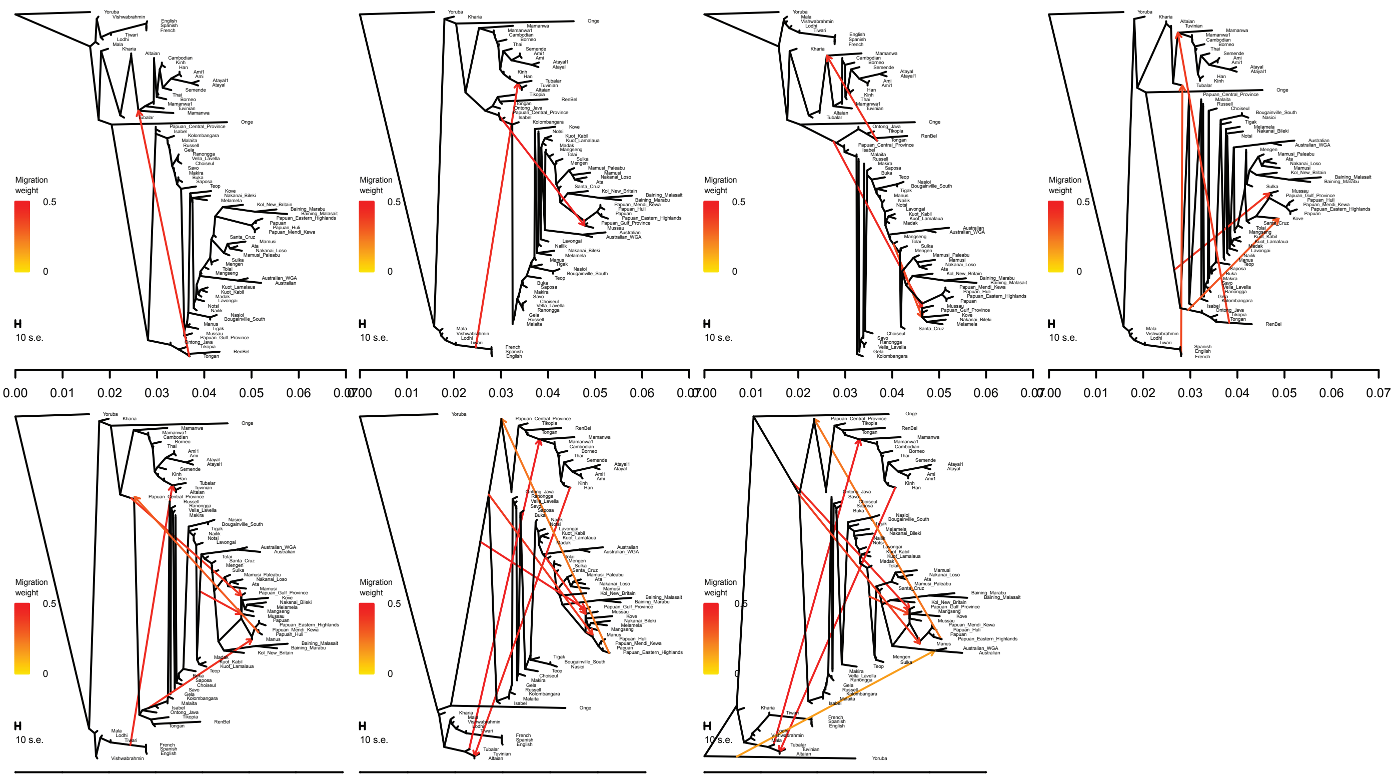

B

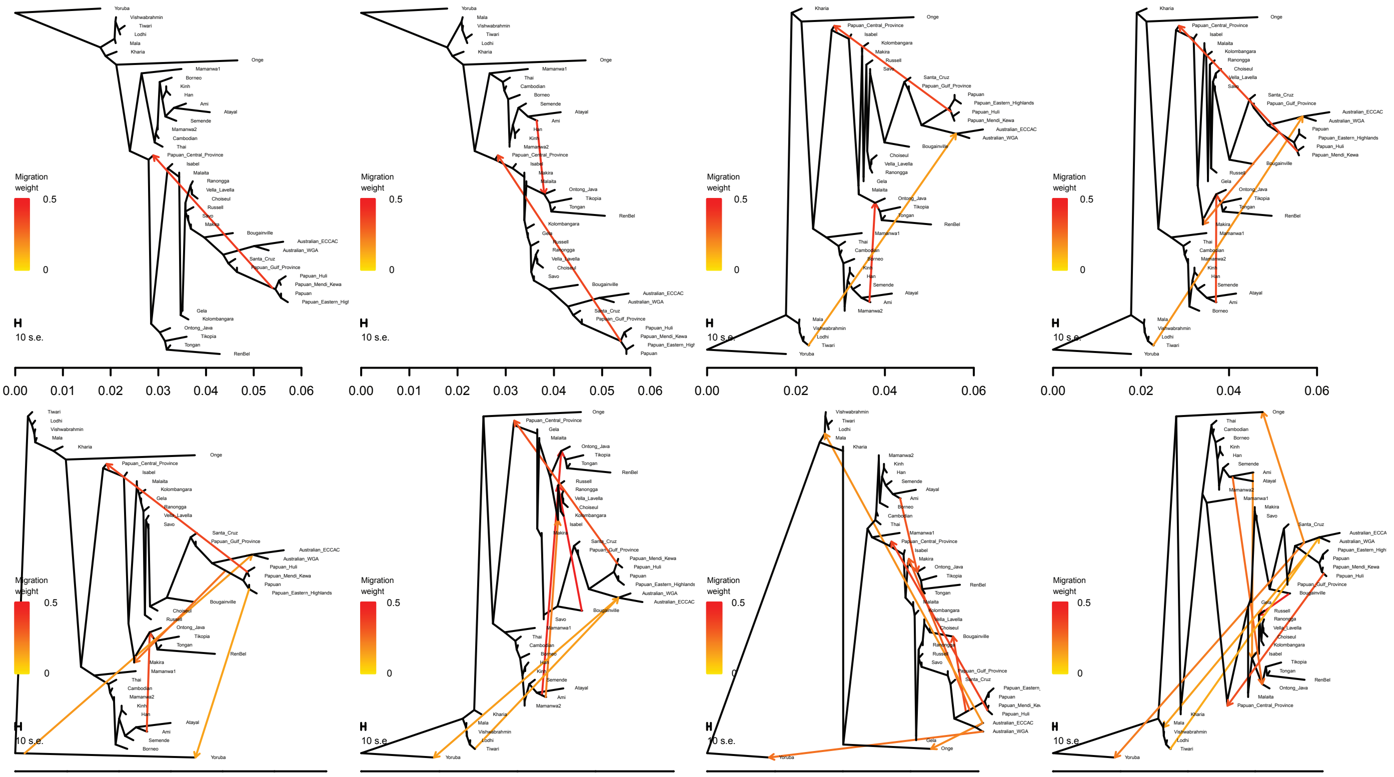

Fig.S11 TreeMix inferred maximum-likelihood tree with (A) seven migration edges for the full data-set (B) eight migration edges for the dataset which excludes samples from the Bismarcks.

figS12

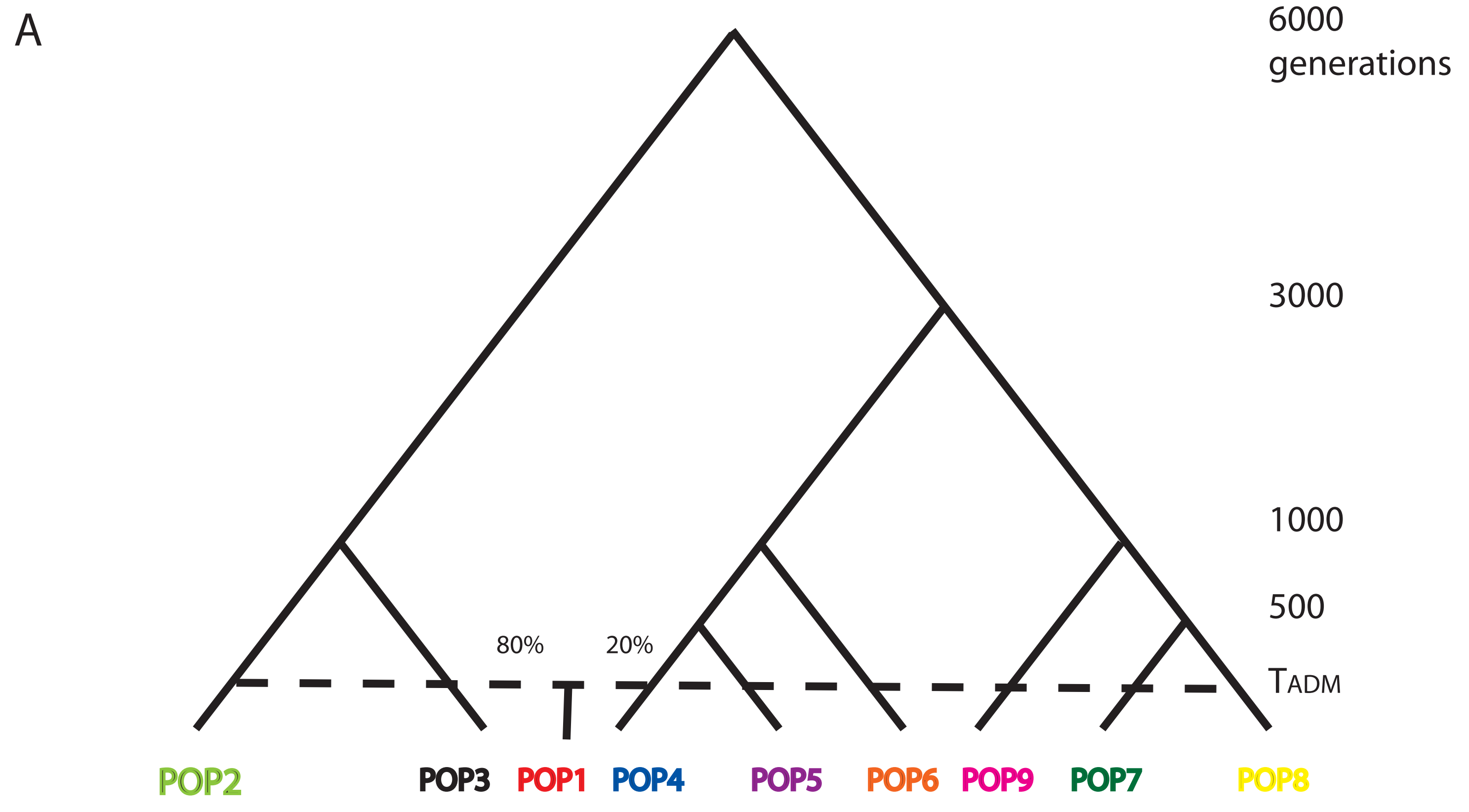

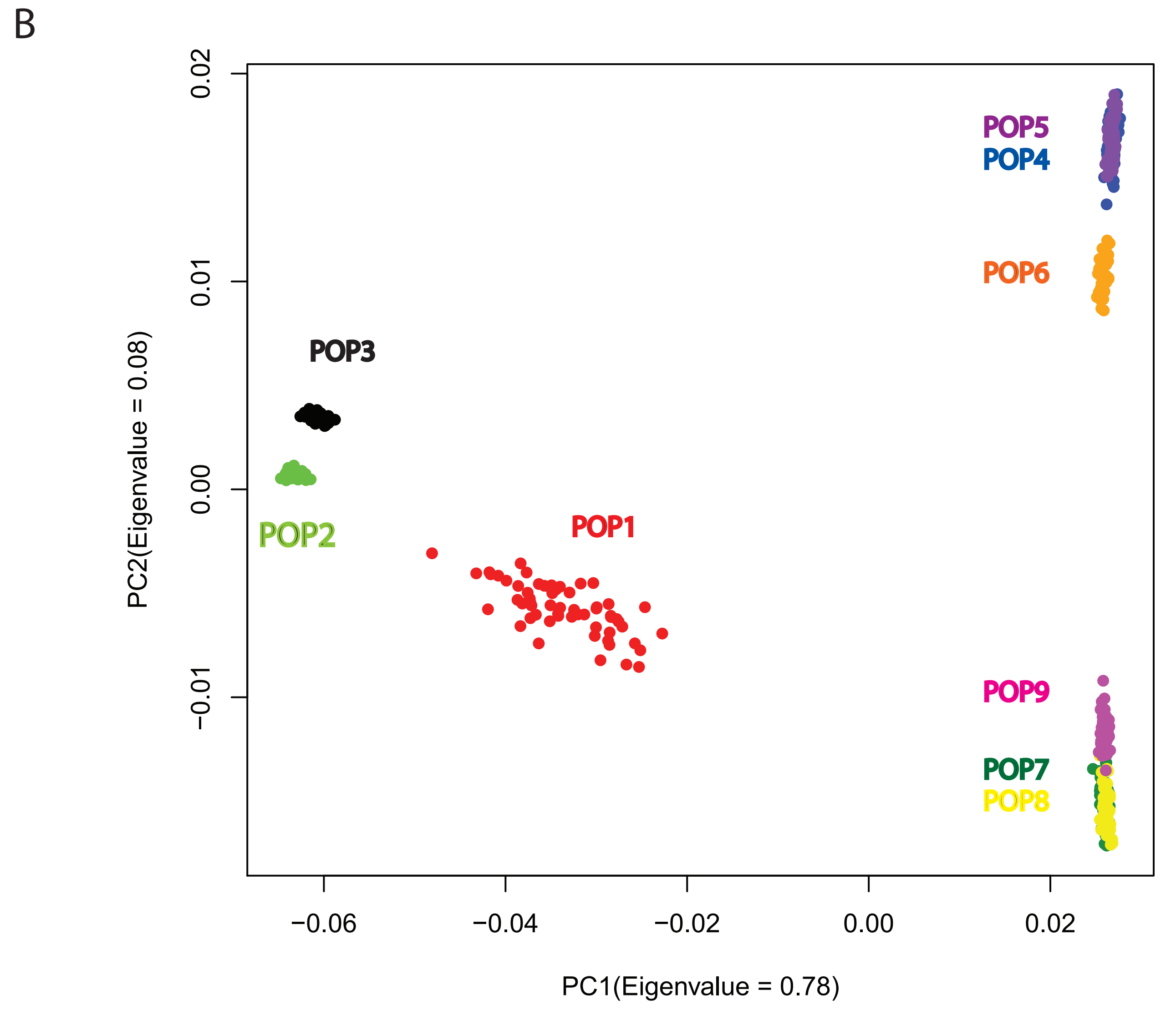

Fig.S12 MaCS simulations. (A) Simulated population history (B) Results of the PC analysis performed on one of the simulated datasets

figS13

A

Baining Malasait

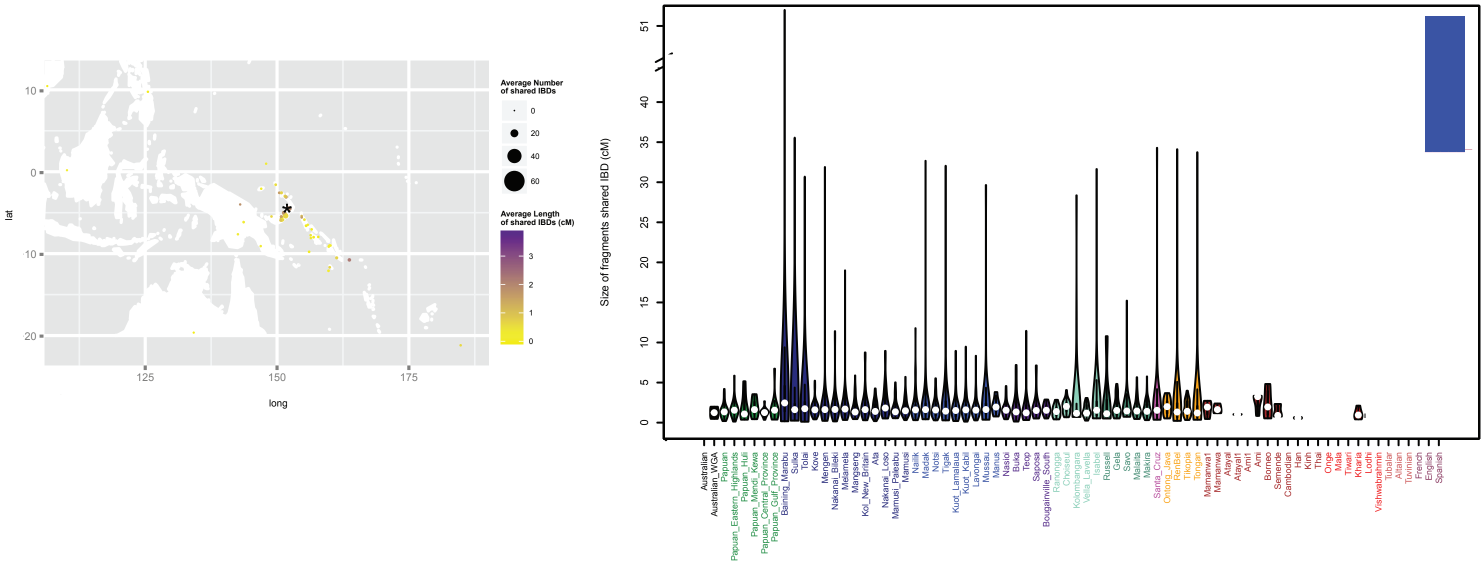

Sulka

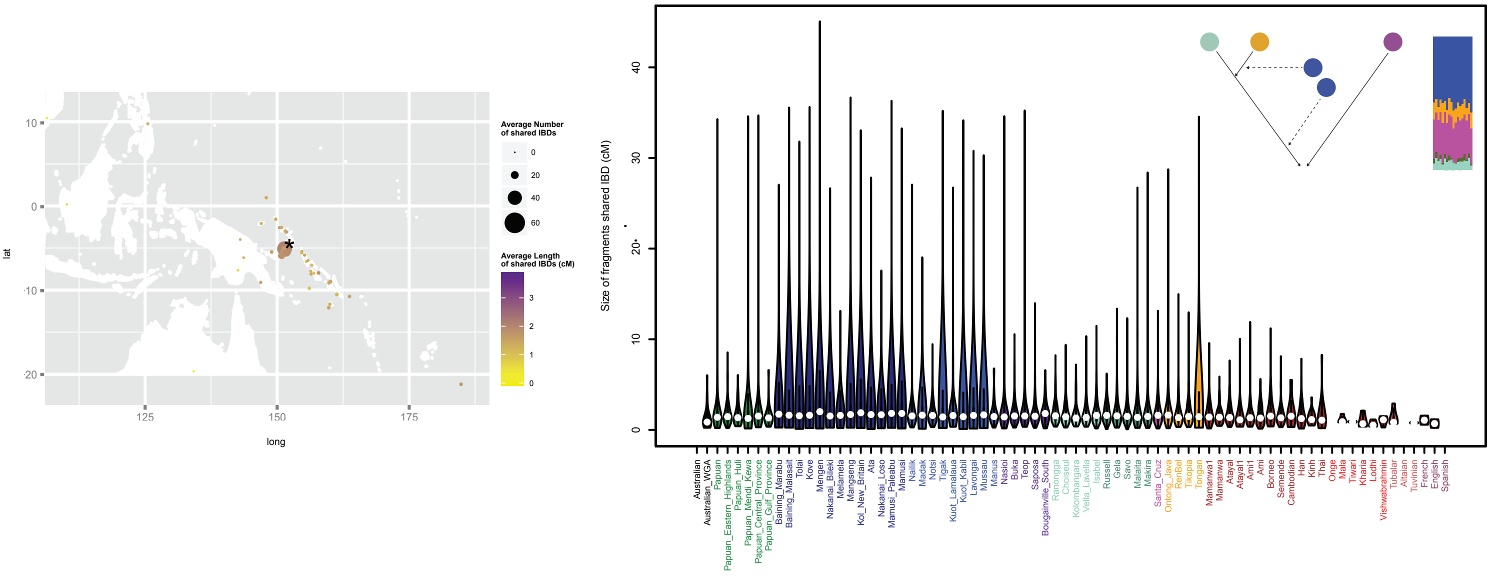

Melamela

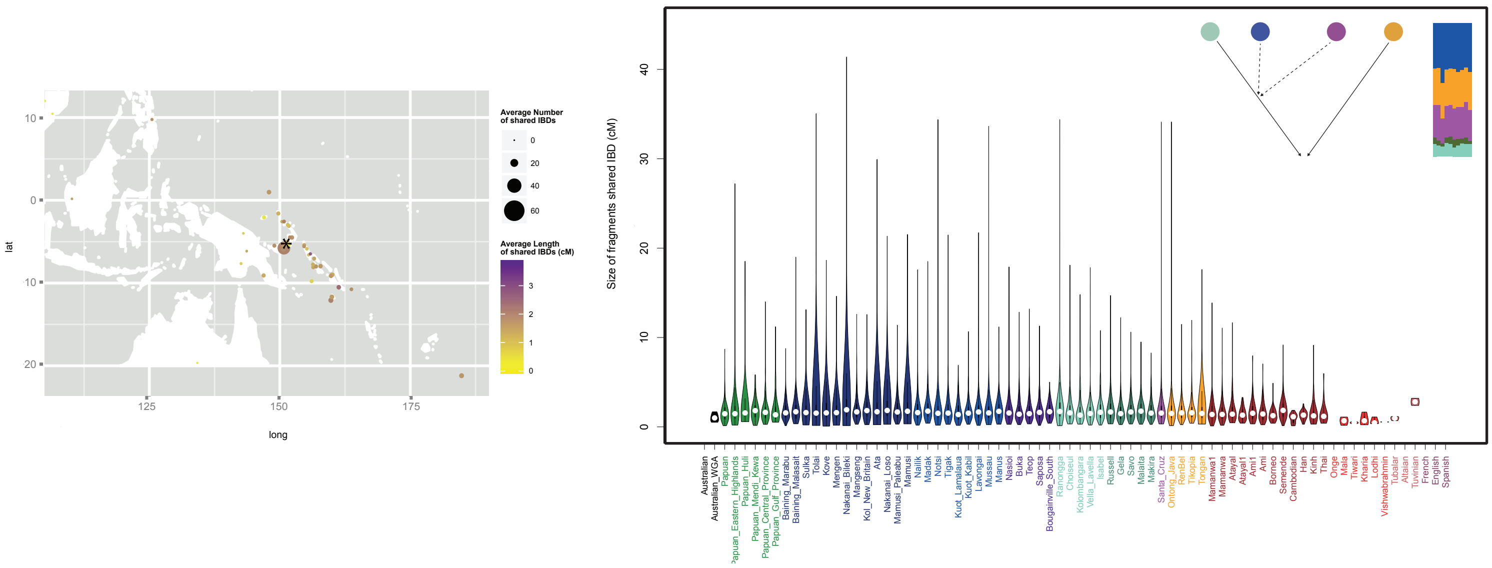

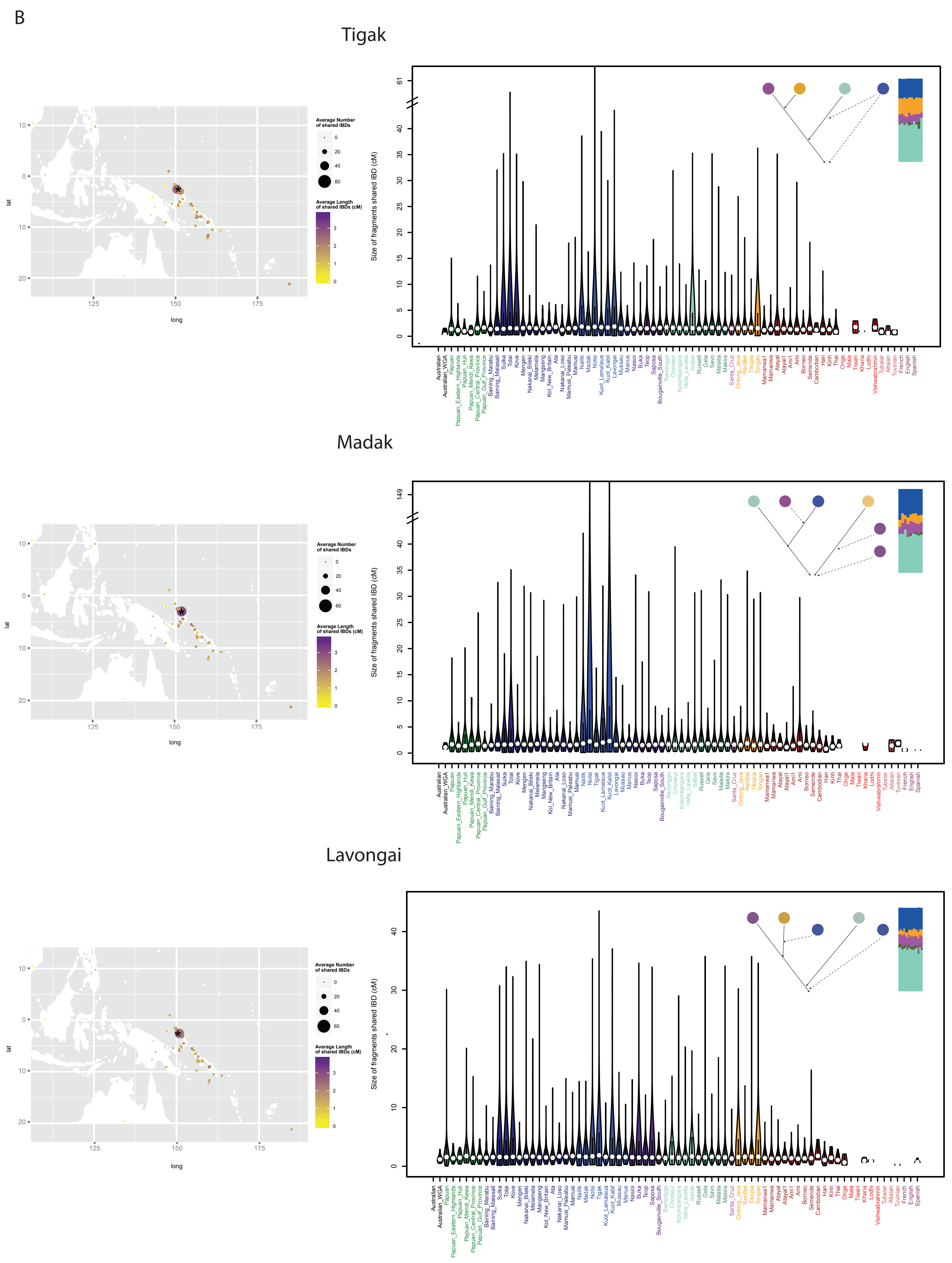
B
Tigak
Madak
Lavongai
Average Number of shared IBDs
Average Length of shared IBDs (cM)
lat
long
Size of fragments shared IBD (cM)
Australian
Australian_WGA
Papuan
Papuan_Eastern_Highlands
Papuan_Huli
Papuan_Mendi_Kewa
Papuan_Central_Province
Papuan_Gulf_Province
Baining_Marabu
Baining_Malasait
Sulka
Tolai
Kove
Mengen
Nakanai_Bileki
Melamela
Mangseng
Kol_New_Britain
Ata
Nakanai_Loso
Mamusi_Paleabu
Mamusi
Nailik
Madak
Notsi
Tigak
Kuot_Lamalaua
Kuot_Kabil
Lavongai
Mussau
Manus
Nasioi
Buka
Teop
Saposa
Bougainville_South
Ranongga
Choiseul
Kolombangara
Vella_Lavella
Isabel
Russell
Gela
Savo
Malaita
Makira
Santa_Cruz
Ontong_Java
Rennell
Tikopia
Tongan
Mamanwa1
Mamanwa
Atayal
Atayal1
Ami1
Ami
Borneo
Semende
Cambodian
Han
Kinh
Thai
Onge
Mala
Tiwari
Kharia
Lodhi
Vishwabrahmin
Tubalar
Altaian
Tuvinian
French
English
Spanish

C

Mussau

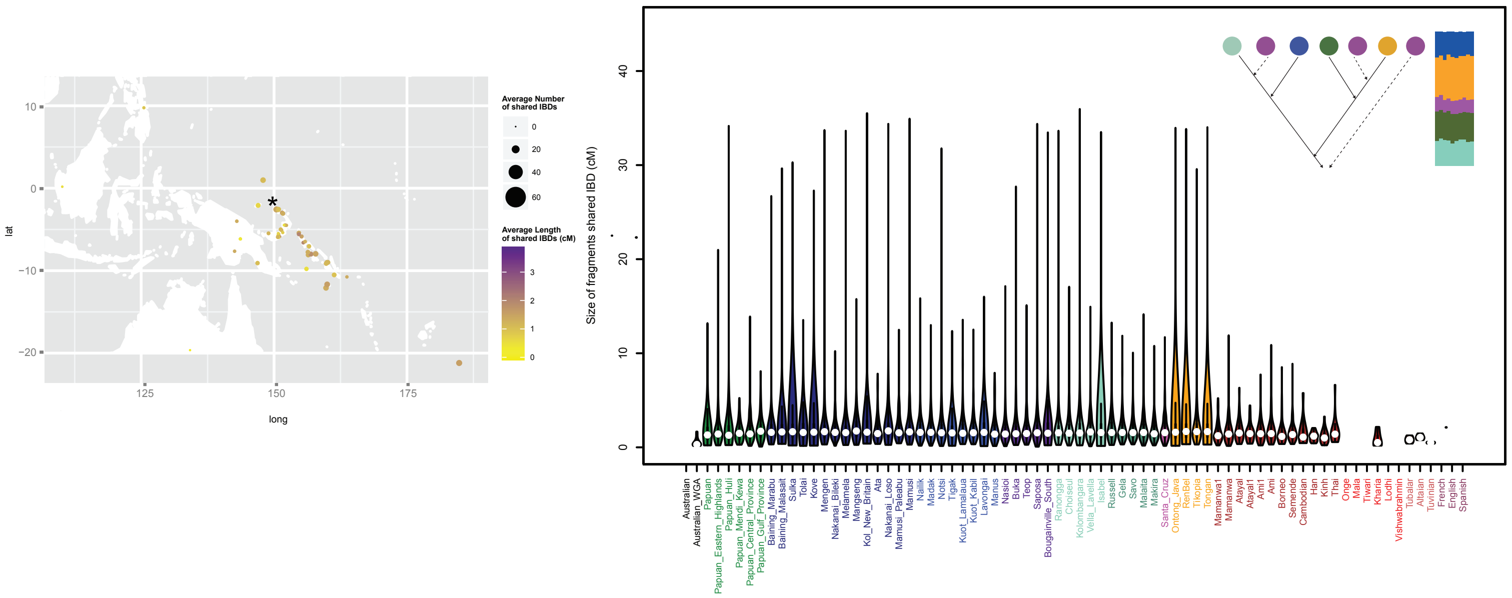

Manus

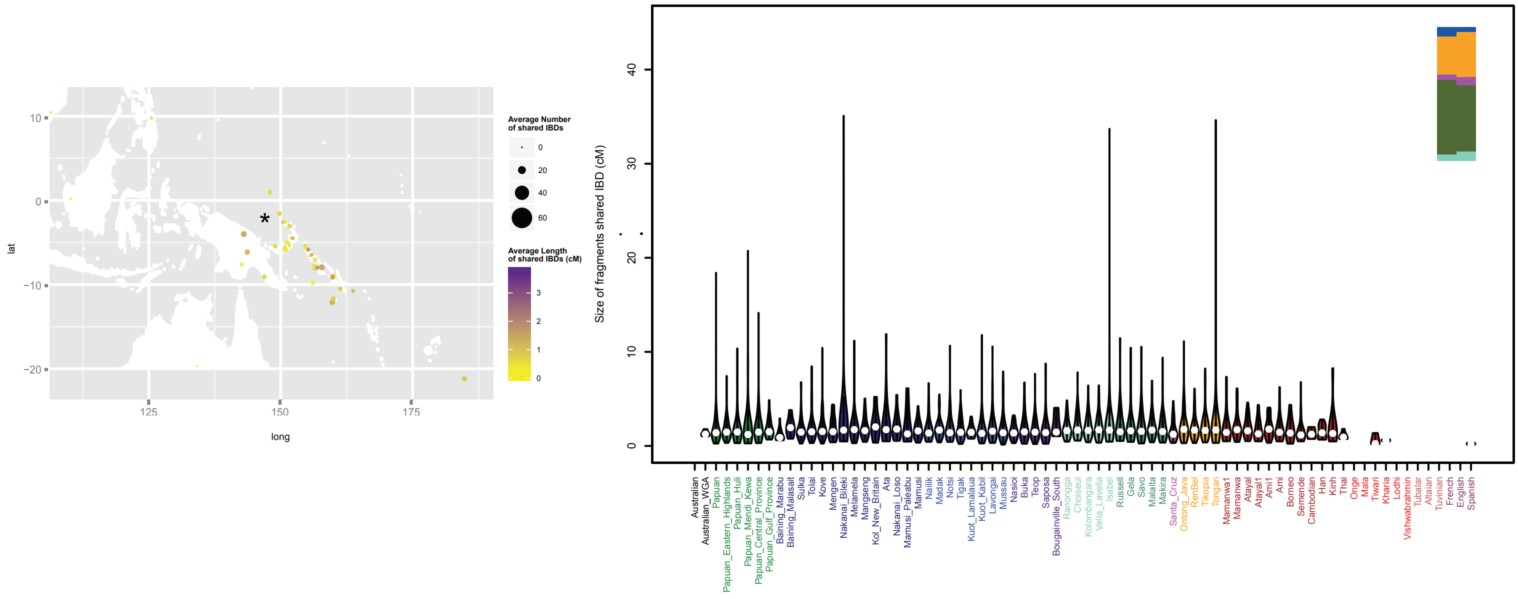

D

Saposa

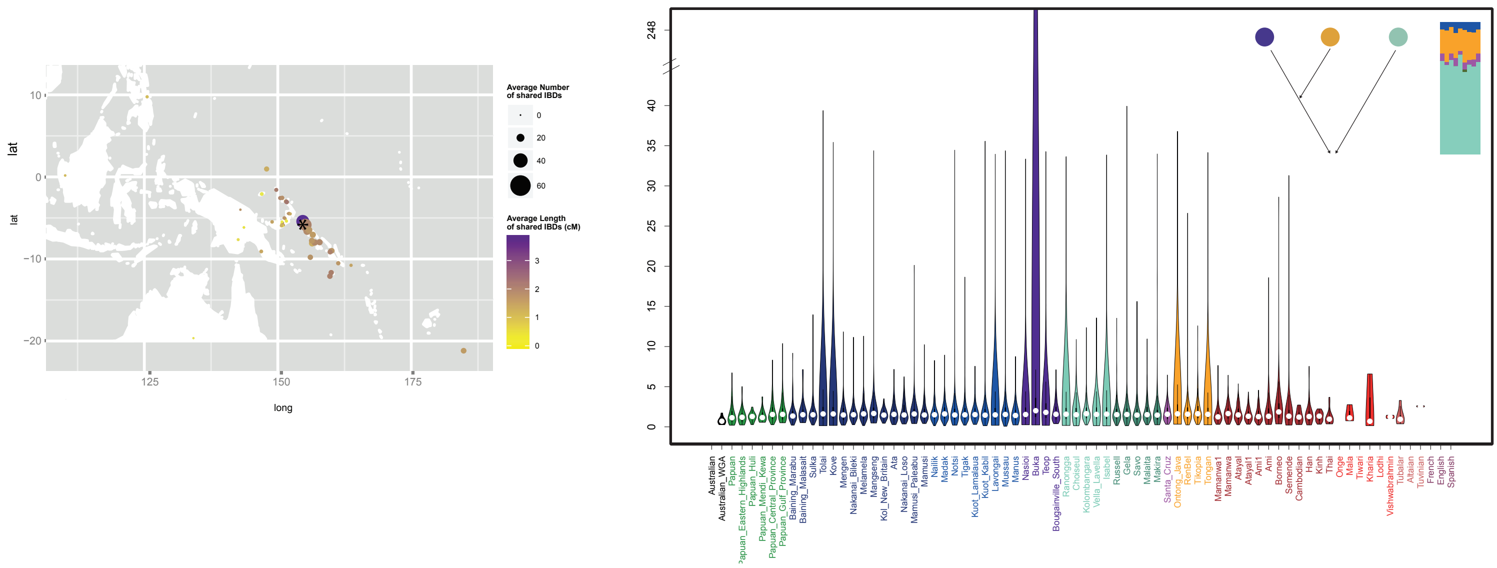

Choiseul

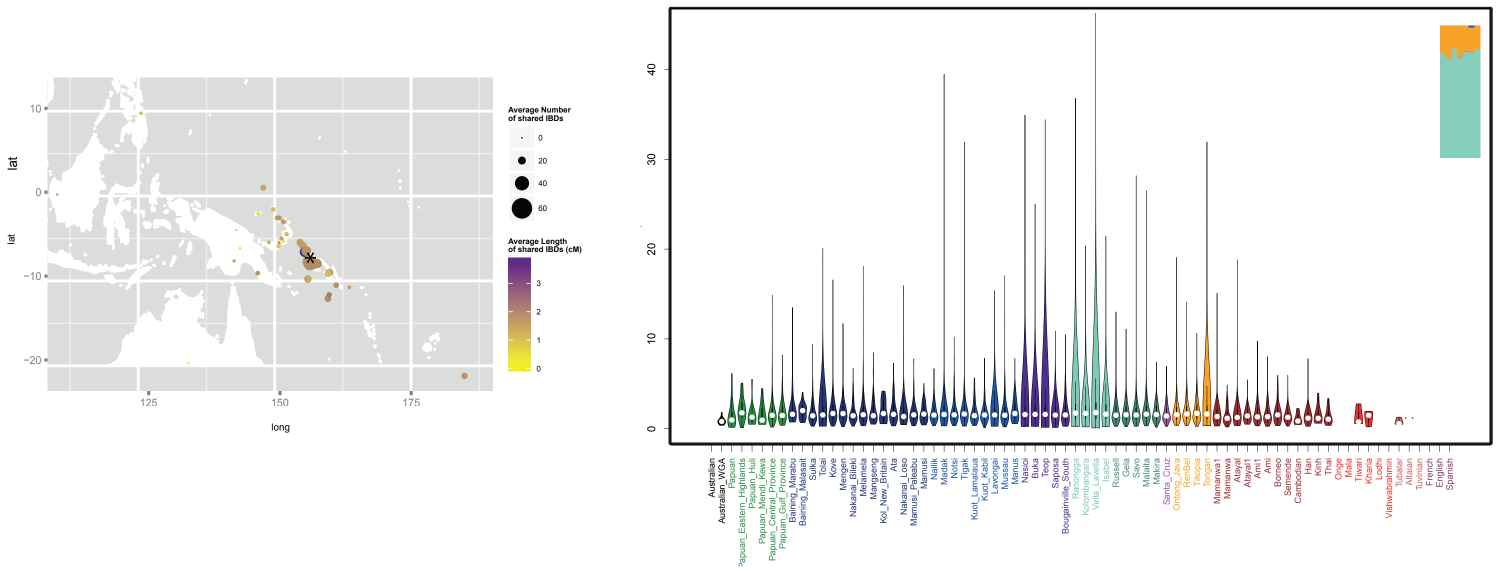

Malaita

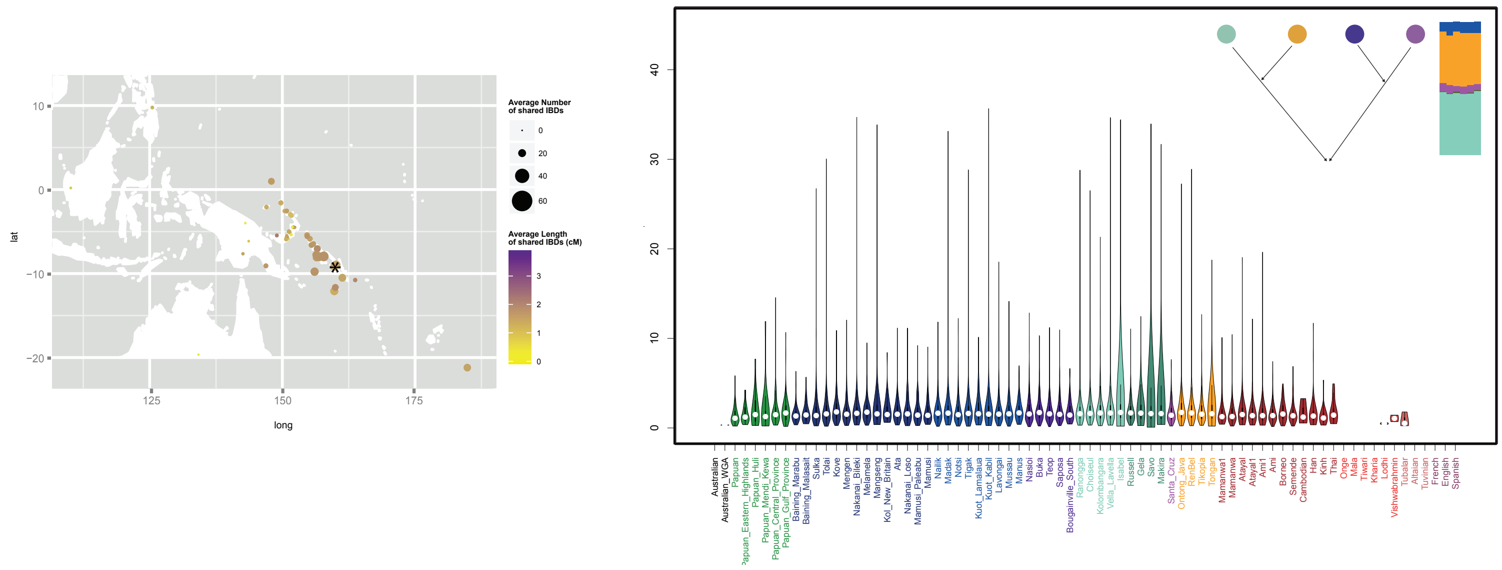

E

Isabel

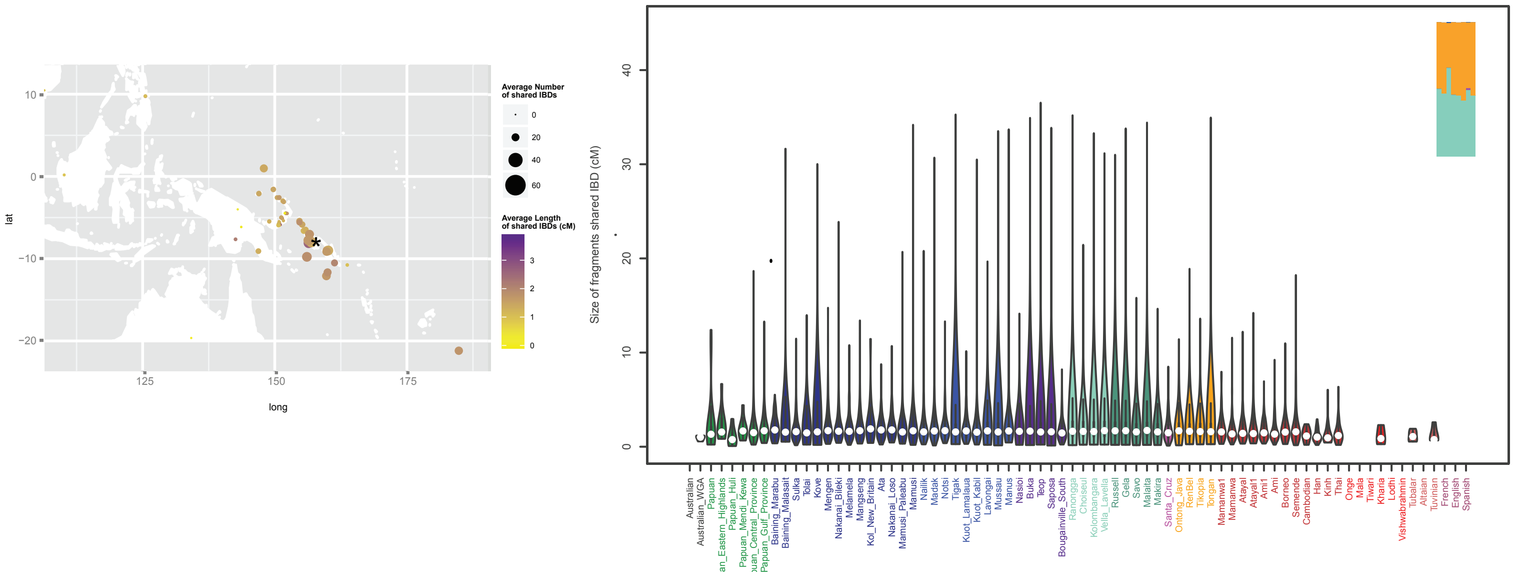

Santa_Cruz

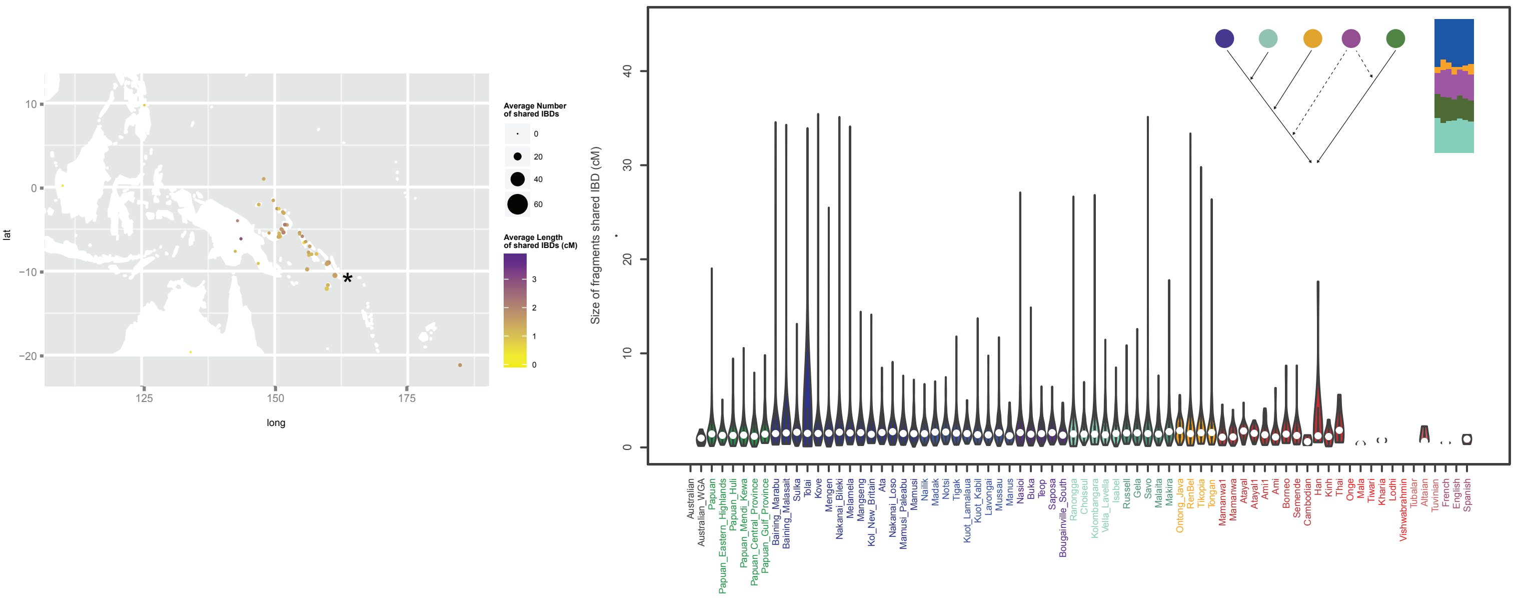

Fig.S13 Recent relatedness based on IBD blocks for representative populations from (A) New Britain in the Bismarcks, (B) New Ireland and Lavongai in the Bismarcks, (C) Mussau and Manus (the northernmost islands in the Bismarck Sea), (D) Bougainville and the Solomon Islands, (E) Isabel and Santa Cruz from the Solomon Islands. For each population, there is: a) a map, which represents the results for the comparison of this population (marked with an asterisk on the map) to each of the other populations in the dataset, with data points placed on the map according to the sampling locations.The size of each circle is proportional to the mean number of IBD segments shared between the population marked with an asterisk and each of the other populations. The color intensity is proportional to the mean length of such shared IBD segments. b) a violin plot, which displays the cumulative distribution of all ancestry blocks, inferred by the IBD analysis for the presented population; the plot captures the total abundance of blocks of each ancestry (x axis) of different genetic lengths in cM (y axis); c) the insets show an excerpt from the plot summarizing results of the ADMIXTURE analysis for the given population at K = 5, and the Admixture History Graph inferring the order of admixture events, if such inference was possible.

figS14

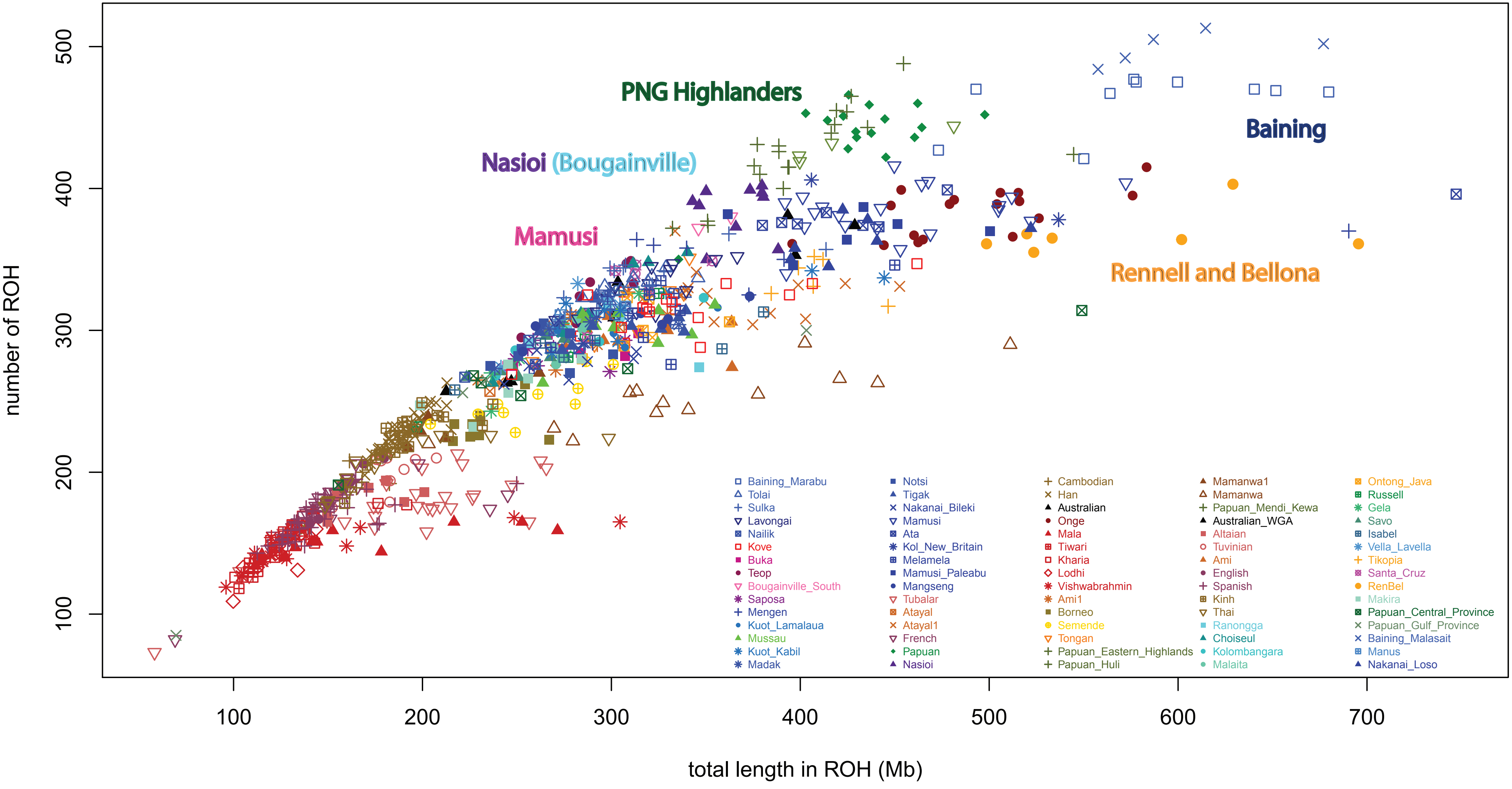

Fig.S14. Total number and length of runs of homozygosity for each individual.

figS15

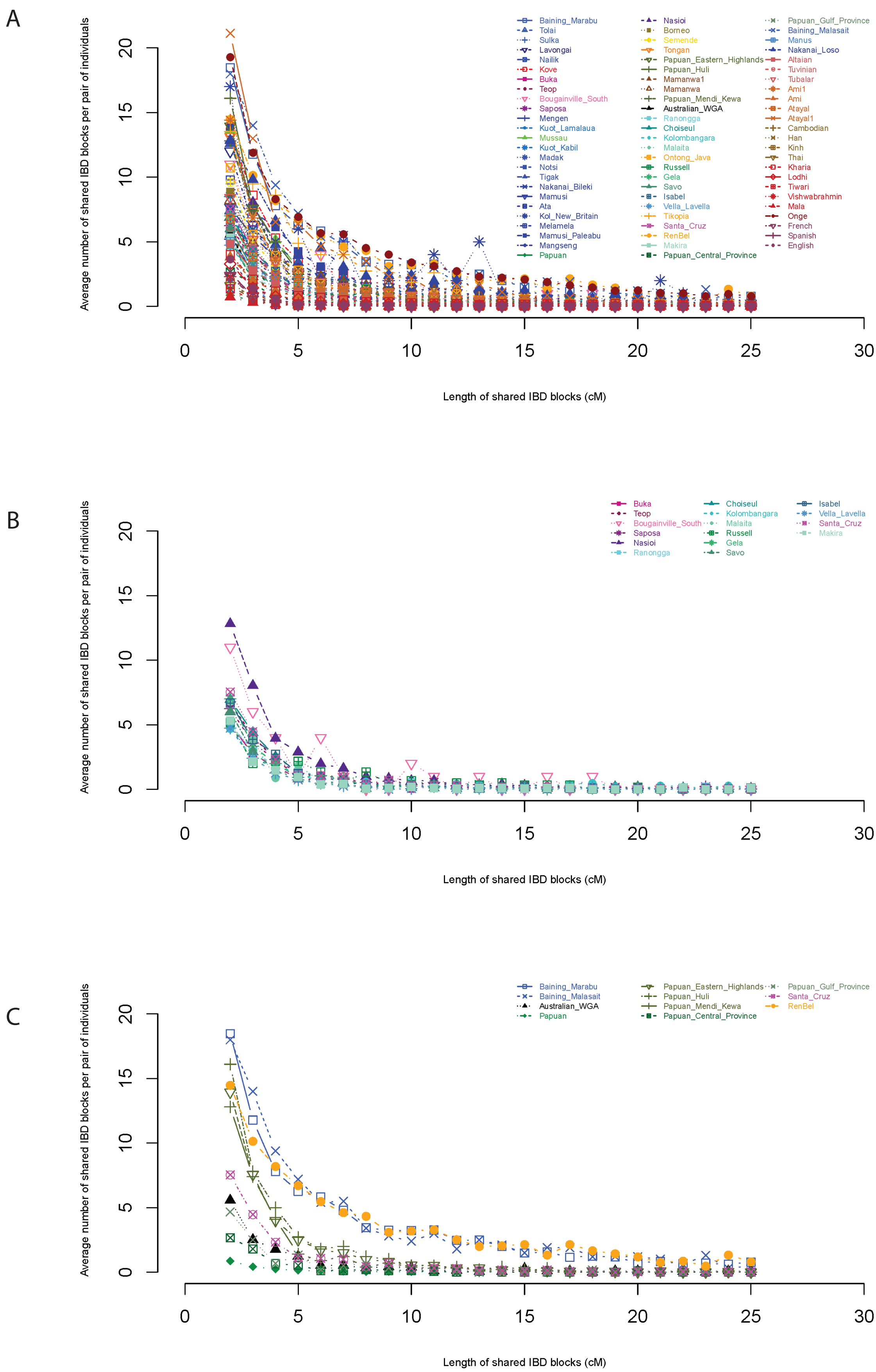

Fig.S15 Analysis of IBD segments shared within populations. The average number of IBD blocks shared per pair of individuals belonging to the same population is plotted against the genetic length of such blocks (blocks smaller than 2 cM were excluded from the analysis). The slow rate of decay in the abundance of short IBD segments in comparison to longer ones suggests founder events in the history of a population (A) Results for the full dataset with all 75 populations included, (B) For ease of visualization only results for the Solomon Islands are plotted, (C) For comparison purposes Santa Cruz is plotted along with the populations which exhibit the slowest rate of decay in abundance of small IBD segments (Papuan Highlanders, Baining from New Britain, and Rennell and Bellona, a Polynesian Outlier) and those with a faster rate of decay (coastal PNG and Australia).

figS16

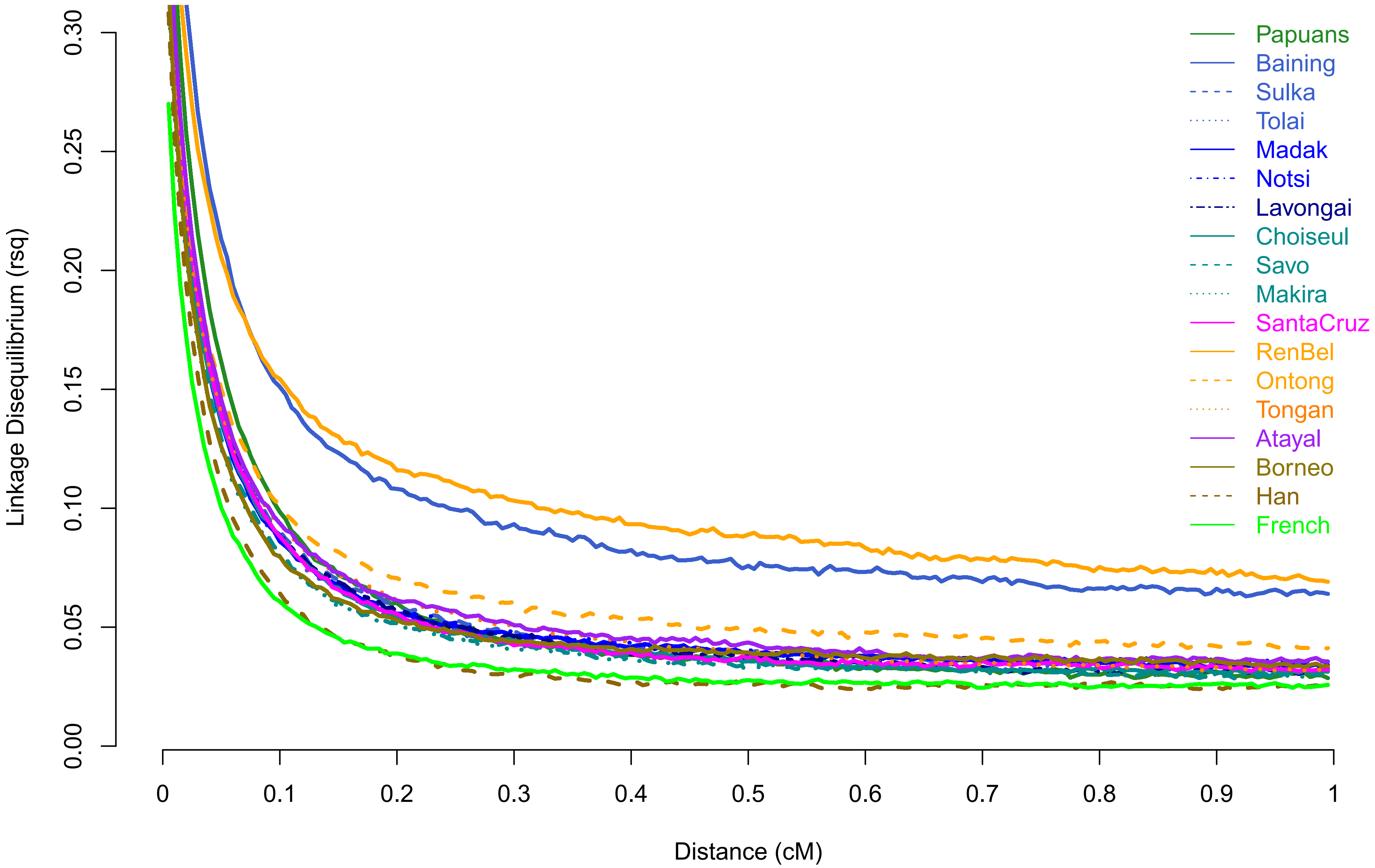

Fig.S16 Patterns of linkage disequilibrium (LD) in select populations. LD was measured for each population and each pair of SNPs within 200 evenly spaced recombination distance categories. The shortest genetic distances between the SNPs are represented on the left and the largest genetic distances on the right.

figS17

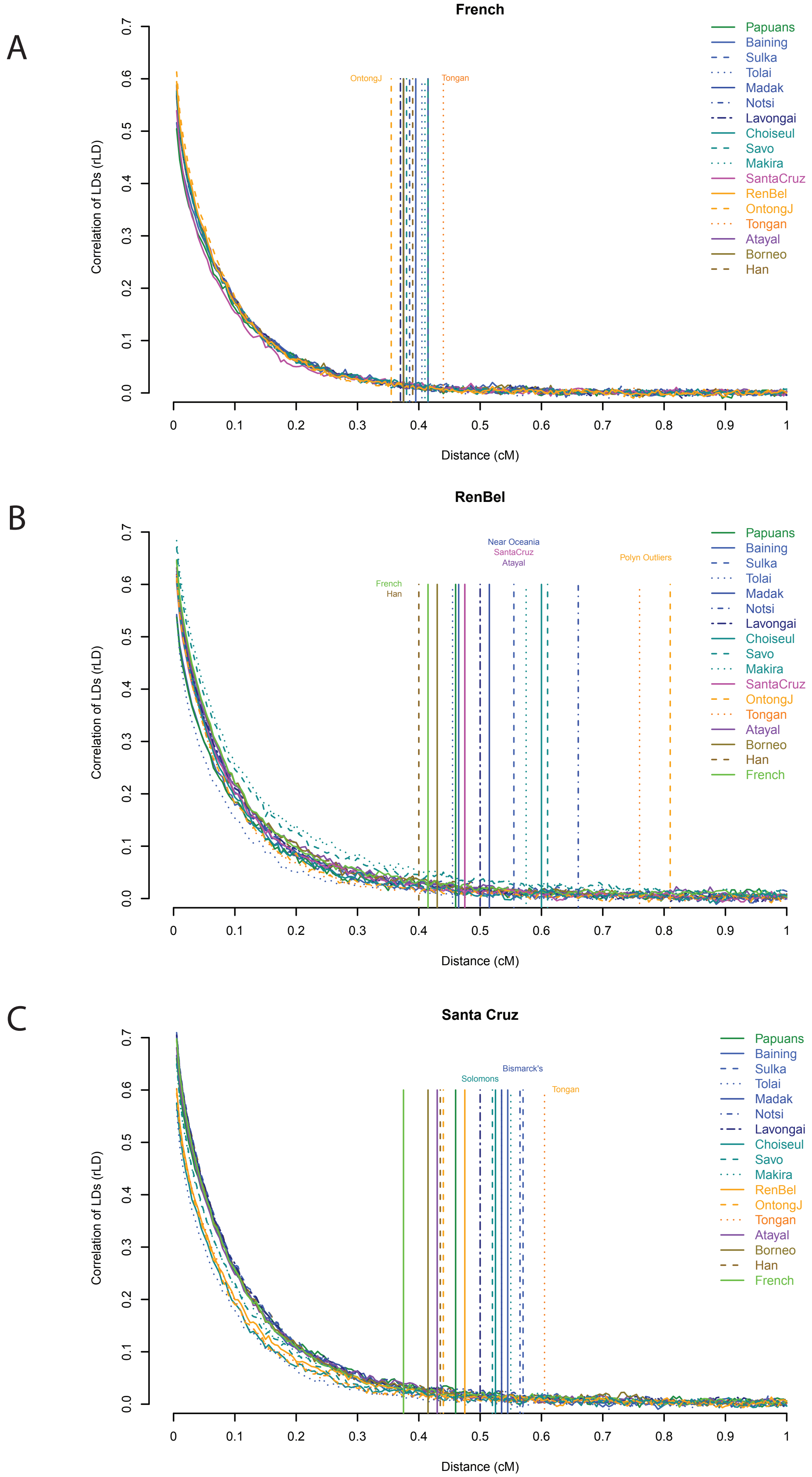

Fig.S17 Correlation of genome-wide LD. Each panel summarizes the decay of LD with genetic distance between (A) French, (B) Rennell and Bellona (Polynesian Outlier), and (C) Santa Cruz, and each of the other populations included in the analysis. For each pairwise calculation, the vertical line denotes the genetic distance at which the correlation becomes less than 0.01.

figS18

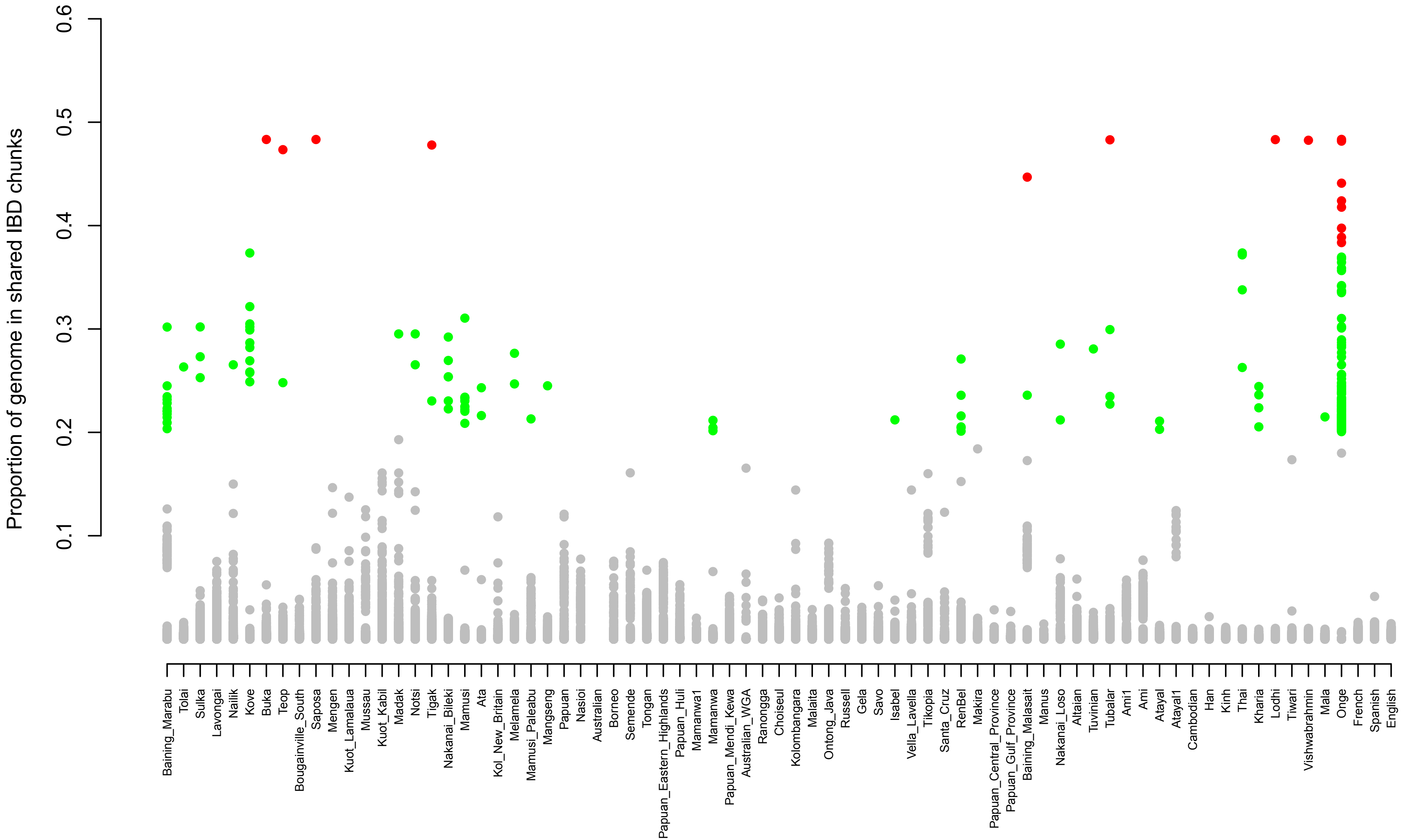

Fig.S18. The proportion of the genome in shared IBD blocks for each pair of individuals. Red = 1st degree relatives; green = 2nd degree relatives, as well as some 3rd and 4th degree relatives

figS19

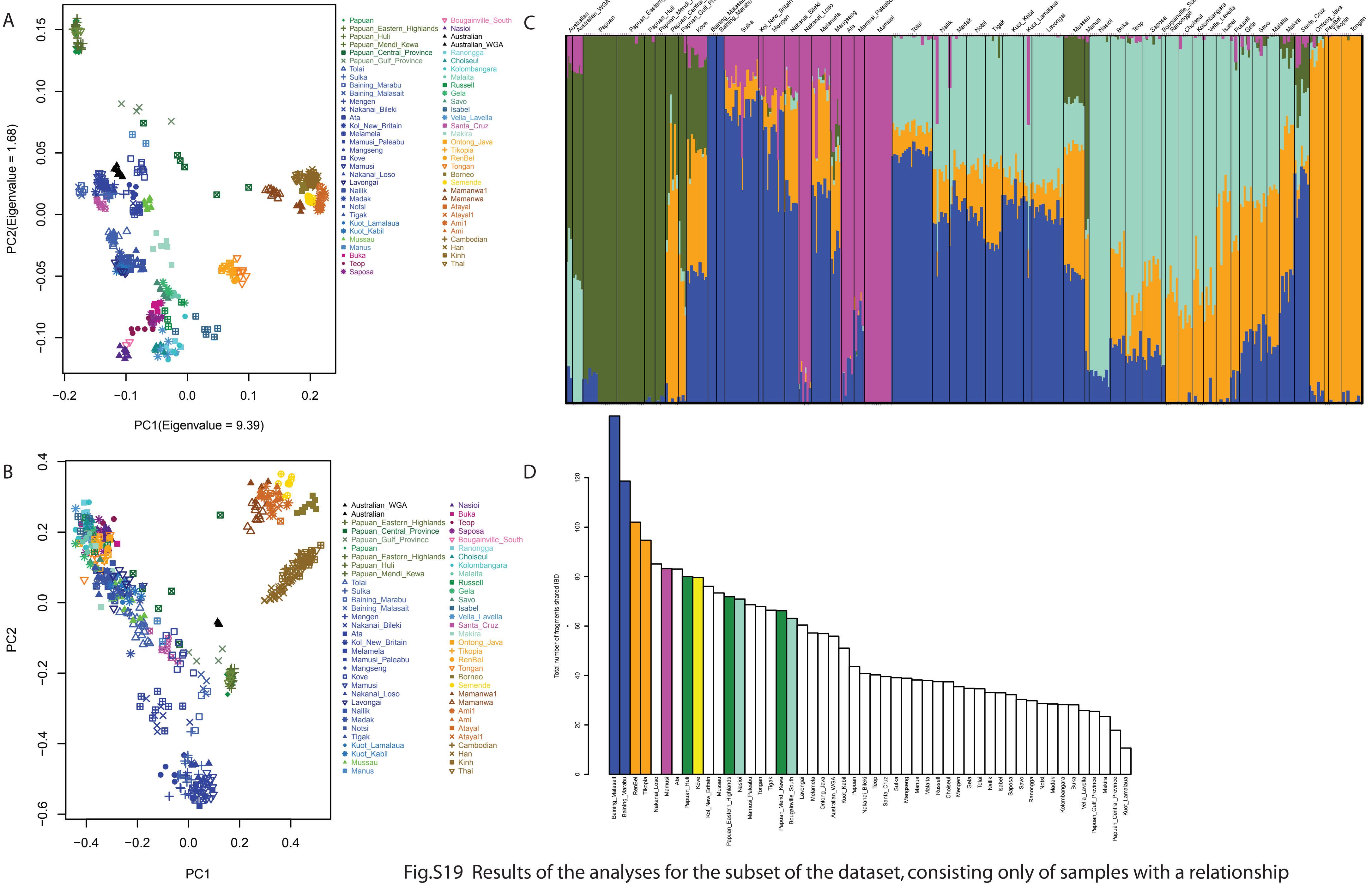

Fig.S19 Results of the analyses for the subset of the dataset, consisting only of samples with a relationship coefficient lower than 0.2 (A) PC analysis based on individual SNPs, (B) PC analysis based on IBD segments, (C) ADMIXTURE for K = 5, (D) within population sharing of IBD segments
